# Characterization of Proteome Variation During Modern Maize Breeding*[Fn FN2]

**DOI:** 10.1074/mcp.RA118.001021

**Published:** 2018-11-08

**Authors:** Lu-Guang Jiang, Bo Li, Sheng-Xue Liu, Hong-Wei Wang, Cui-Ping Li, Shu-Hui Song, Mary Beatty, Gina Zastrow-Hayes, Xiao-Hong Yang, Feng Qin, Yan He

**Affiliations:** From the ‡MOE Key Laboratory of Crop Heterosis and Utilization, National Maize Improvement Center of China, China Agricultural University, Beijing 100094, China;; §College of Biological Sciences, China Agricultural University, Beijing 100094, China;; ¶Agricultural College, Hubei Collaborative Innovation Center for Grain Industry, Yangtze University, Hubei 434000, China;; ‖BIG Data Center, Beijing Institute of Genomics, Chinese Academy of Sciences, Beijing 100101, China;; **DuPont Pioneer, Johnston, IA 50131

**Keywords:** iTRAQ, RNA SEQ, Gene Expression, Statistics, Transcriptional Regulation, eQTL, pQTL, Proteome, Temperate Adaptation, Zea mays

## Abstract

The success of modern maize breeding has been demonstrated by remarkable increases in productivity with tremendous modification of agricultural phenotypes over the last century. Although the underlying genetic changes of the maize adaptation from tropical to temperate regions have been extensively studied, our knowledge is limited regarding the accordance of protein and mRNA expression levels accompanying such adaptation. Here we conducted an integrative analysis of proteomic and transcriptomic changes in a maize association panel. The minimum extent of correlation between protein and RNA levels suggests that variation in mRNA expression is often not indicative of protein expression at a population scale. This is corroborated by the observation that mRNA- and protein-based coexpression networks are relatively independent of each other, and many pQTLs arise without the presence of corresponding eQTLs. Importantly, compared with transcriptome, the subtypes categorized by the proteome show a markedly high accuracy to resemble the genomic subpopulation. These findings suggest that proteome evolved under a greater evolutionary constraint than transcriptome during maize adaptation from tropical to temperate regions. Overall, the integrated multi-omics analysis provides a functional context to interpret gene expression variation during modern maize breeding.

## Abstract (precis)

The integrated multi-omics analysis provides insights into variation at different gene expression levels during the adaption of modern maize from tropical to temperate regions. Population-specific proteome variation mirrors genetic variation better than mRNA levels, and a class of cis-QTLs were identified that regulate protein abundance with little or no effect on mRNA levels. Thus, the discordance between protein and mRNA levels indicates far greater evolutionary stability of proteome during modern maize breeding.

## Graphical Abstract

**Figure:**
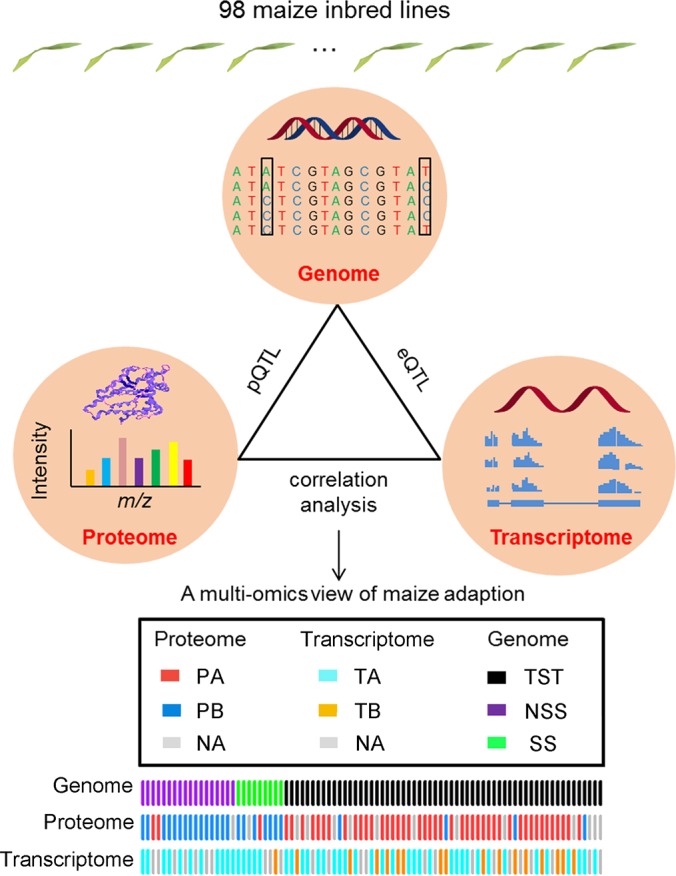

- Protein level cannot be reliably interpreted from RNA-level at a population scale.
- Proteome may evolve under a greater evolutionary constraint than transcriptome.
- *cis*-pQTLs provide an important resource for posttranscriptional regulation studies.

Maize was domesticated from teosinte (*Zea mays* ssp. *Parviglumis*) about 9000–10,000 years ago in southwestern Mexico, a mid- to low-land tropical growing environment (1–3). After a long period of selection and improvement by farmers, the cultivation of maize experienced a marvelous spread across the world from tropical geographic origin to the temperate regions, with a remarkable increase in productivity for this crop (4). The temperate-tropical division of maize germplasms remains in all crop-growing continents today (5, 6). When coped with extensively varying temperate conditions such as photoperiod, disease susceptibility and temperature, maize adapted extraordinarily well (7–9). The importance of genomic and transcriptomic changes contributing to the advantageous adaption process has been a central tenet of recent studies (10–12). However, till now, we know almost nothing about how proteome amended in correspondence to genomic and transcriptomic changes underlying the maize temperate-tropical division.

Assessment of mRNA levels has revealed substantial differences in transcriptome across inter- or intra-species in plant and led to the identification of putatively adaptive changes in transcript expression (13, 14). Conventionally, measurements of variation in mRNA levels are assumed to be good proxies for divergence in protein levels (15–19). However, there are multifaceted mechanisms by which protein expression may be regulated without changing mRNA levels (20, 21). If transcript and protein expression levels are hypothetically uncoupled, mRNA levels may evolve under reduced constraint because variations at the transcript level could be compensated or buffered at the protein level (21–23). To date, the accordance of transcript and protein level at a population scale has not been interpreted in any plant species. Therefore, we still lack the fundamental knowledge about what is the extent of the evolutional constraint during plant evolution. Such protein-centric mechanism would be only addressed by integrative evaluation of genetic, mRNA and protein-level variations at a population scale (24–26).

To decipher the molecular mechanisms underlying maize diversity, variation and genetic loci responsive for mRNA expression levels (eQTL)^1^ have been extensively analyzed (27, 28). In addition, previous studies have also interpreted the effect of genome variation on levels of endogenous metabolites, such as amino acids, fatty acids, hormones, flavonoids as well as vitamins, etc., leading to the identification of a series of metabolic quantitative trait loci (mQTLs) (29, 30). Although some recent mapping efforts have elucidated genetic determinants of protein levels (pQTL) in mouse, yeast and human (31–36), genome-wide mapping of protein abundance in plants has been missing.

Here, we used high-throughput high-resolution mass spectrometry (MS) to quantify ca. 3000 proteins in an association panel including 98 maize inbred lines. Then we integrated the quantitative proteomics with RNA sequencing data to reveal a comprehensive view of the accordance of mRNA and protein levels during the adaption of maize from tropical to temperate regions.

## EXPERIMENTAL PROCEDURES

### 

#### 

##### Experimental Design and Statistical Rationale

Maize inbred lines were germinated in soil and transplanted in cultivation boxes (35 × 20 × 10 cm, length × width × depth), which were filled with 3 kg of enriched soil (turf to vermiculite in a ratio of 1:1) and maintained under 25 °C in a 16-h-light/8-h-dark condition. At two-week-old, the second true leaves from 10 seedlings were harvested in the morning and pooled to make one biological replicate. The sampling processes for all the inbred lines were conducted within two hours. Samples were ground into fine powder in liquid nitrogen and divided into two parts, which were subjected to proteomic and RNA-seq analysis. All the inbred lines used in RNA-seq analysis include two independent biological replicates, whereas seven representative inbred lines used in iTRAQ proteomic analysis include two independent biological replicates and the other 91 inbred lines with one biological replicate.

##### Protein Extraction

For protein extraction, the ground samples were suspended in cold acetone (−20 °C) containing 10% trichloroacetic acid (TCA) for 2 h. After centrifugation (20,000 × *g*) at 4 °C for 30 min, the supernatant was carefully discarded. The precipitate was rinsed with 80% of cold acetone for three times and dissolved in lysis buffer (8 m urea, 30 mm HEPES, 1 mm PMSF, 2 mm EDTA, and 10 mm DTT), followed by sonication for 5 min. After centrifugation (20,000 × *g*) at 4 °C for 30 min, the supernatant was collected and reduced with 10 mm DTT at 56 °C for 1 h. Then 55 mm iodoacetamide (IAM) was added to block the cysteines for 1 h in the dark. The mixture was precipitated with a 4-fold volume of cold acetone for 3 h at −20 °C, followed by centrifugation at 20,000 × *g* for 30 min. The resulting pellet was redissolved in 0.5 m triethylammonium bicarbonate (TEAB) buffer with 0.1% SDS, and sonicated for 3 min. Finally, the samples were centrifuged at 20,000 × *g* for 30 min. The supernatant was collected and quantified with the Bradford assay using BSA (Bio-Rad, Hercules, CA) as the standard.

##### Trypsin Digestion and iTRAQ Labeling

For each sample, 100 μg proteins were digested with 3.3 μl trypsin (1 μg/μl, Promega, Madison, WI) at 37 °C for 24 h. Then, an additional 1 μl of trypsin was added, and the sample was digested again for 12 h. The digests were vacuum-dried, dissolved in 30 μl 0.5 m TEAB and mixed with 70 μl isopropanol. The digested peptides were labeled with 8-plex iTRAQ reagents (Applied Biosystems, Foster City, CA) according to the method provided by the manufacturer. A total of fifteen 8-plex kits were performed including biological replicates.

##### SCX Fractionation and LC-MS/MS

The pooled peptides were vacuum-dried and reconstituted with strong cation exchange (SCX) buffer A (10 mm KH_2_PO_4_ in 25% acetonitrile), after that the pH was adjusted to 3.0 with phosphoric acid. The peptides were eluted at a flow rate of 1 ml/min with the following gradient of buffer B (10 mm KH_2_PO_4_ and 2 m KCl in 25% acetonitrile, pH 3.0): 0% for 40 min, 0–5% over 1 min, 5–30% over 20 min, 30–50% over 5 min and maintained for 5 min, and 50–100% over 5 min and maintained for 10 min. Then, the fractions were desalted with a strata-X C18 column (Phenomenex, Torrance, CA) according to the manufacturer's instructions and vacuum-dried. The peptide mixtures were dissolved in buffer A (0.1% formic acid in water) and loaded onto a Dionex Ultimate 3000 Nano LC system connected to a Q-Exactive mass spectrometer (Thermo Fisher Scientific, Waltham, MA). Peptides were separated with a reversed-phase C18 analytical column (100 × 75 mm, 5 μm, 300 Å; Agela Technologies, Tianjin, China), using a gradient of 5–80% buffer B (0.1% formic acid in acetonitrile) over 45 min at a flow rate of 400 nL/min. A full mass spectrometry (MS) scan (350–2000 *m*/z) was acquired at a resolution of 70,000 (at 200 *m*/*z*). Information on peptides and peptide fragments *m*/z were obtained using the following conditions: an AGC target value of 3e^6^; number of scan ranges of 1; higher collision energy dissociation (HCD) fragmentation; micro-scans of 1; an isolation window of 2 *m*/*z*, ion fragments were detected in the Orbitrap at a resolution of 17,500 (at 200 *m*/*z*); and the electrospray voltage applied was 1.8 kV.

##### LC-MS/MS Data Analysis

For protein identification, the raw mass data were processed using Proteome Discoverer 1.3 (Thermo Fisher Scientific) and MASCOT 2.3.01 (Matrix Science, London, UK) against the Uniprot_*Z. mays_*86922_20141226.fasta (86922 sequences, download December 26th, 2014) protein database with the following parameters: carbamidomethylation of cysteine residues as a fixed modification; trypsin was chosen as the enzyme with one missed cleavage allowed; iTRAQ 8-plex modification of the N terminus, K and Y, Gln → Pyro-Glu of the N terminus and oxidation of methionine were set as variable modifications; monoisotopic mass was chosen; the peptide MS and MS/MS tolerances were set at 15 ppm and 20 mmu, respectively. Finally, the same set of MS spectral data was searched as above, but against the maize decoy database to calculate a false discovery rate (FDR) using the Proteome Discoverer program. Then, high-confidence peptides were obtained by setting a FDR threshold of 1% at the peptide level. Two criteria were used for the protein quantitation: (1) only unique peptides were used for quantitation; (2) the median ratio (inbred line/reference line) of all peptides mapping to the same gene was considered as the relative protein level.

##### MRM Analysis

Approximately 2 μg of digested peptides were analyzed on a TripleTOF 6600 System (AB SCIEX, Concord, NH) equipped with a nano LC system (Shimadzu Corporation, Kyoto, Japan). The data was then searched using ProteinPilot^TM^ Software5.0 (AB SCIEX, Framingham, MA) against the maize Uniprot database with the following parameters: Iodoacetamide as Cys alkylation, trypsin as the digestion enzyme, sample type as identification and Biological as ID focus. Skyline 4.1.0.11796 (McCoss Lab, University of Washington, WA) was used to establish the MRM transition list. Then the selected transitions were adopted to survey the protein digests from the individual inbred lines. All 12 MRM samples were performed on a QTRAP6500 mass spectrometer (AB SCIEX) equipped with an ekspert nano LC 425 system (Eksigent, part of AB SCIEX). The mobile phase consisted of solvent A (0.1% aqueous formic acid in water) and solvent B (100% acetonitrile with 0.1% formic acid). Peptides were separated on an eksigent column (150 × 0.075 mm, 5 μm, 200 Å, Eksigent) and then eluted using a gradient of 5–30% solvent B for 30 min and 30–80% solvent B for 15 min at 400 nL/min. For the QTRAP6500 mass spectrometer, ion spray voltage of 2400 V, curtain gas at 35, collision gas at high, interface heater temperature at 150 °C, entrance potential at 10, and MRM transitions were monitored using unit resolution for Q1 and Q3 quadrupoles (supplemental Table S1). MRM assay development met applicable criteria for Tier 3 MRM assay (37).The MRM raw data generated on QTRAP6500 were input into Skyline 4.1.0.11796 (MacCoss Lab). To ensure correct peak detection and integration, manual inspection was performed for further filtering the peptides. The median values for the MRM peak areas of the target peptides in B73 line were set as normalization references, and the relative protein abundance of each target protein was calculated by averaging the all corresponding peptides.

##### RNA-seq Data Processing and Reads Mapped

Total RNA was isolated using TRIzol reagent (Biotopped, Beijing, China). Polyadenylated mRNA was purified using the Dynabeads® mRNA Purification kit (Invitrogen, Carlsbad, CA) and library construction was performed with the TruSeq RNA Sample Preparation Kit (Illumina, San Diego, CA) following manufacturer's instructions. Sequencing was conducted on the Illumina HiSequation 2500 system with TruSeq SBS v3 reagents (38). For each sample, the resulting sequences were trimmed based on quality scores and mapped to the B73 maize genome (ZmB73_RefGen_v4) by using Hisat2 (version 2.0.5) (39) with parameters: -dta, -score-min L,-0.6,-0.6. Only uniquely mapped reads were taken for the subsequent analysis.

##### RNA Expression Analysis

The FPKM values (fragments per kilobase of transcript per million mapped reads) of RNAs in each sample were calculated using Stringtie (version 1.3.4d) (40) with parameters: -G -e -B -o -A. Information regarding maize reference genome annotation was obtained from Ensembl Plants (ftp://ftp.ensemblgenomes.org/pub/plants/release-38/gtf/zea_mays/).

##### MapMan Categorical Enrichment Analysis

Based on the Pearson's correlation coefficients between mRNA and protein, a Mapman categorical enrichment analysis using the Kolmogorov-Smirnov test was used to assess the concordance between protein and mRNA variation in the context of the biological function of the gene products.

##### Coexpression Networks Construction and Preservation

To adjust for population stratification, a linear regression of protein and mRNA levels on population label (NSS, SS, TST) was performed and the residuals were normalized by transforming the quantiles of the residual values to their respective quantiles of a N (0, 1) distribution (32). Then coexpression networks for both mRNA and protein were built according to the methods of WGCNA (Weighted gene correlation network analysis) (41) in an R environment. The soft power thresholds (β) was set to 9 to satisfy the scale-free topology criterion for protein and mRNA networks (42). Adjacency matrices were built with argument type “signed” using biweight midcorrelation (bicor). Next, a topological overlap matrix (TOM) was generated to measure the gene dissimilarity (43) and used as input for identifying gene modules based on average linkage hierarchical clustering (44). Each gene corresponded to a branch of the hierarchical dendrogram and modules was clustered by cutting branches referring to a cutoff height (45). In our analysis, the minimum module size was set to 30 genes. The first principal component of each module was denoted as module eigengene (ME) and modules whose eigengenes were more than 0.85 of correlation were remerged. Module definitions in the protein network were then imposed to the mRNA network. Each gene's module membership (MM) for a given module was then estimated to measure correlations between each gene and each ME. For the final module characterization, all the genes with MM values above 0.4 were assigned to that module, leaving some genes assigned to multiple modules. This thresholding procedure allowed us to measure module overlap with any other lists using the hypergeometric distribution (46). Although the value represents one of many possible sets of module-thresholding parameters, the results were relatively robust to changes in module size. Finally, we used a variety of strategies including qualitative and quantitative means to measure module preservation. First, we assessed the significance of module overlap between genes in corresponding protein and mRNA modules using hypergeometric test. Second, we used a permutation test procedure (“modulePreservation” function, nPermutations = 200, corFnc = “bicor”) implemented in the WGCNA R package (41), which produces a summary preservation *Z*-summary score. *Z*-score above 10 means module highly preserved, *Z*-score in between 2 and 10 is weak to moderate preservation, whereas *Z*-score below 2 indicates no preservation (47).

##### Gene Set Enrichment Analysis

FuncAssociate 3.0 (48) was used for all gene set enrichment analysis in this study. The ensembl gene identifiers were used for the enrichment tests. Because *Zea mays* is not yet supported by FuncAssociate, the maize GO annotation file was downloaded from agriGO (http://bioinfo.cau.edu.cn/agriGO/download/item2term_82) (49, 50). The gene list for background input was explicitly defined as the set of genes that could potentially be included in the query set. We defined significant enrichments as GO terms with an odds ratio greater than 2 and adjusted *p* value < 0.05. *p* value adjustment was performed using a permutation method to account for the overlap between the GO terms.

##### Identification of Proteomic and Transcriptomic Subtypes

A total of 1375 proteins and 1339 mRNAs, which were variably expressed among the samples with a MAD (median absolute deviation) value in top 50% of the dataset, were used for subtype identification. A consensus clustering was conducted according to the methods implemented in ConsensusClusterPlus R package (51, 52). Briefly, perturbations of the original data were simulated by resampling techniques. Clustering algorithm was applied to each of the perturbed data sets and the consensus among the multiple runs was assessed and summarized in a consensus matrix. Visual inspection of the consensus matrixes and the corresponding summary statistics (for example, area under the curve) was used to determine the optimal number of clusters as previously described (53). The parameters used were set as clustering algorithm = hierarchical clustering; clustering metrics = (1-Pearson correlation) distance and average linkage; n resamplings = 1000; proportion of samples and proteins used in each resampling = 80%; k tested = from 2 to 8 (54). Silhouette analysis was carried out to identify “core” samples (R package: cluster) (55). Only members with a positive silhouette value were retained for further analysis as highly representative samples of their subgroup assignment (56).

##### Subtype Signature Identification

To identify protein signatures for individual proteomic subtypes, we compared protein expression in one subtype against the residuals. A two-sided Wilcoxon rank-sum test was used to call significant difference. The *p* values were corrected for multiple testing using the Bonferroni method and the statistical significance was determined based on a corrected *p* value of less than 0.05. A leave-one-out cross validation (LOOCV) (57–59) was performed to evaluate the generalizability of the proteomic subtypes and their signature proteins. Briefly, one of the 73 samples was set aside and the remaining 72 samples were used to identify protein signatures and train a nearest shrunken centroid classifier for the proteomic subtypes using the R package pamr (54, 60). Then, the trained classifier was applied to the set-aside sample. This was repeated 73 times for all samples and the cross-validation error rate was calculated. We obtained a low misclassification error rate of 2.7%, suggesting good generalizability of the proteomic subtypes and their signature proteins.

##### Self-Organizing Maps for Integrative Analysis of Gene Expression and Genomic Subpopulation

A self-organizing maps (SOMs) analysis (61, 62) was used to explore the relationship between two gene expression level measurements and genomic subpopulation. To avoid skewing distance calculation because of difference in scale and variance, the mRNA and protein expression across the genomic subpopulations were converted to percentiles using the empirical cumulative distribution function for each level as previously described (26). For mRNA expression and protein levels, we used the median expression value across all inbred lines. After conversion to percentiles, we retained genes with all six measurements: mRNA expression in NSS, SS, and TST; protein levels in NSS, SS, and TST. The modified kohonen R package (63, 64) was used for the SOM training. The total number of neurons and the x-y dimensions of the SOM were defined as previously described (64). We used a toroid with a hexagonal grid for the map. The learning rate of the SOM was reduced linearly from 0.05 to 0.01 during training, and each data point was presented to the map 100 times. Given that the initial configuration of the map is random, the resulting maps would be different from each other. To catch the best possible initial configuration, we repeated the SOM training 1000 times with different random number generator seeds. We chose the random number seed that minimized mean distances of the data points to the codebook vectors of the winning units in the SOM. The best SOM according to this criterion was achieved by setting R session random number generator seed to 784. After iterations of the SOM for 1000 times, there was minimal divergence with the best SOM showing less than 0.98% difference in error than the average error of the nonoptimal SOMs. We then clustered the codebook vectors of the 88 units in the SOM using affinity propagation clustering (65) as implemented in the apcluster R package (66). We calculated the input pairwise similarity measures using the negative squared distances. The exemplars for affinity propagation clustering were initialized by setting the *q* parameter to 0.1.

##### Identification of QTL and Enrichment Analysis

All genetic association analyses were performed using a linear mixed-effects model (67, 68), considering population structure and kinship in TASSEL 5.2 (69). Only genes detected in at least 50% of the samples for both mRNA and protein measurements were analyzed. To identify *cis*-QTL, the genotypes of SNPs located in the corresponding gene region ± 200 kb and with MAF > 5% were tested, resulting in an average of 170 SNPs (ranging from 1 to 802) analyzed for each gene. The Benjamini-Hochberg (BH) test was applied to control FDR (70) at level α = 0.05 (BH rejection threshold: *p* < 1.98 × 10^−4^ for *cis*-pQTL; *p* < 3.47 × 10^−4^ for *cis*-eQTL). For *trans*-pQTL mapping, each of genome-wide 466,435 SNPs was tested for association with quantification of the 2,657 proteins. To deal with multiple testing problem, 46,345 markers that were in approximate linkage equilibrium with each other were identified from PLINK (71) based on SNP pruning (plink -file data -indep-pairwise 50 50 0.1) (72, 73). Under a Bonferroni correction, the suggestive *p* value cutoff to control the genome-wide type I error rate was set to 10/(46,345 × 2,657) = 8.12 × 10^−8^. To eliminate false-positive associations introduced by the linkage disequilibrium (LD) structure, the most significant SNP within a corresponding LD region (r^2^ ≥ 0.1) was defined as a QTL for the tested gene (28).

A likelihood ratio test (LRT) was conducted to identify SNPs significantly associated with the protein levels taking mRNA expression and population structure as covariates (conditional model) to account for effects fully mediated by transcription. Specifically, we compared two linear models: *Y* = α*X* + β*p* + γ*M* + ε and *Y* = β*p* + γ*M* + ε. In the two models, *Y* is protein expression level, *X* is the SNP genotype, *P* is the population structure, α is the protein-specific effects, β is the population structure effects, *M* is the mRNA expression level, and ε is the random effects. Then, the protein-specific QTLs statistical significance was set based on a Bonferroni corrected *p* value threshold of 0.05. SNPs located in the corresponding c*is*-genes (± 200 kb) were categorized according to their position annotation (exonic, intronic and extragenic) in the maize B73 genome (ZmB73_RefGen_v4). SNPs located in the exonic region were further categorized into CDS, 5′ UTR and 3′ UTR. For each gene, the annotations of SNPs were based on the transcript which has the longest coding sequence. We used a hypergeometric test to evaluate the distribution of the *cis*-QTLs for different genomic and functional annotations. For distinct annotations, we restricted each of the tests to SNP-gene pairs matching an appropriate “background.” For instance, when testing for enrichment of *cis*-pQTLs in CDS, we considered all CDS SNPs located in all the 267 *cis*-genes.

## RESULTS

### 

#### 

##### Proteome Landscape of 98 Maize Inbred Lines

We conducted isobaric tags for relative and absolute quantitation (iTRAQ)-based quantitative mass spectrometry to quantify protein expression variation in 98 maize inbred lines. Those lines represent part of an association panel, which was widely used in previous studies (72–74). The samples can be divided into three subpopulations and one mix group, which are termed by SS (*n* = 9), NSS (*n* = 18), TST (*n* = 60) and MIXED (*n* = 11), respectively (75, 76). SS and NSS subpopulations are of temperate origin, and TST subpopulation is of tropical or subtropical origin. MIXED subpopulation encloses inbred lines which were not accurately assigned into the three subpopulations based on the phylogenetic analysis (75, 76). In the experimental setup, 8-plex iTRAQ was used, and the B73 line was taken as the universal control in every 8 samples to quantify the relative protein abundance for the other seven samples. This approach yielded 15 8-plex iTRAQ experiments in total (*see Experimental Procedures for details*). To ensure adequate sample size and statistical power, proteins unable to be quantified in less than half of lines were removed. Eventually, a total of 2750 proteins were defined with unique Ensembl gene ID and used for the further analysis (supplemental Table S2).

**Fig. 1.**
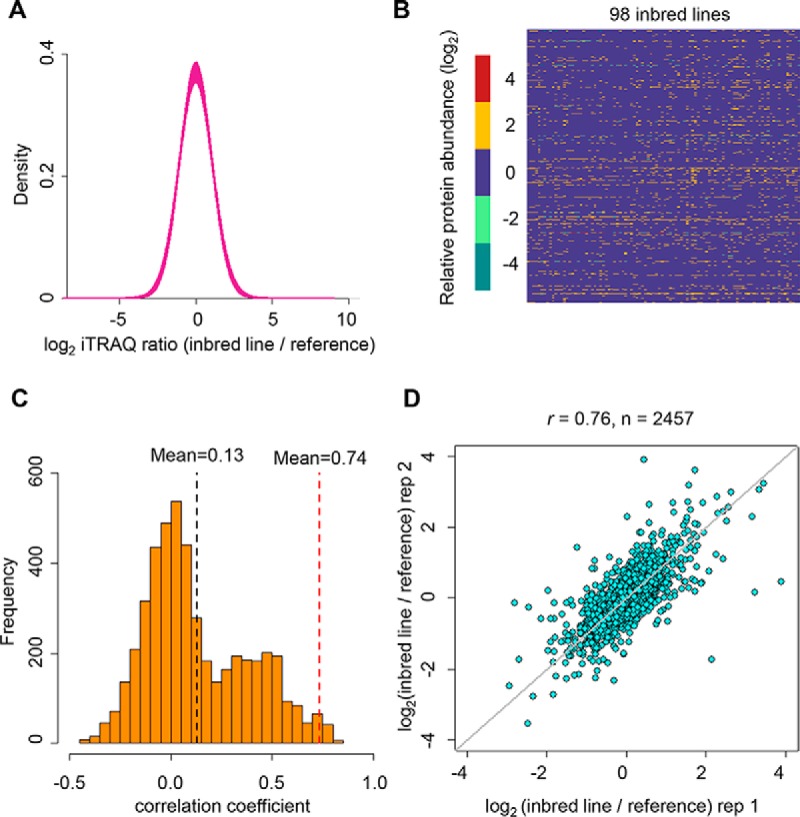
**Proteome quality control.** *A*, Distribution plot of log_2_-transformed proteome ratios (individual inbred line *versus* B73) for all the 98 samples. *B*, Heat-map of log_2_-transformed proteome ratios (individual inbred line *versus* B73) for all the 98 samples. *C*, Distribution plot of the correlation coefficients between pairs of non-replicates with a mean correlation coefficient of 0.13 (black dashed line). We then calculated the correlation coefficient between the seven replicate lines (mean value of 0.74, red dashed line). We carried out 100,000 permutations to judge whether the replicate line correlations were signiicantly higher than non-replicates. (*p* < 1 × 10^−5^). *D*, The Spearman's correlation of two replicates in a representative inbred line CIMBL157. Each point denoted a protein.

To assess the quality of the proteomics data, we evaluated the unimodality distribution for each sample. The density plot of log_2_-transformed ratios of protein abundance (inbred lines *versus* B73) in each sample conformed to a unimodal distribution (Fig. 1*A*) and no significant tailing was observed in the unimodality test (supplemental Fig. S1*A*), indicating that none of evident degradation or contamination happened in the samples. In addition, the heat map using the log_2_-transformed ratios from 941 proteins which were detected in all the 98 samples didn't show irregularity with unusually low or high abundance of those proteins when compared with that in B73 (Fig. 1*B*). Moreover, an independent biological replicate was carried out for 7 representative inbred lines, and we observed that the correlation coefficients (Fig. 1*C* and 1*D*; supplemental Fig. S1*C*) or R^2^ for simple linear regression (supplemental Fig. S1*B*) among non-replicate samples was significantly less than that of biological replicates (permutation test, *p* < 1 × 10^−5^). Taken together, these results demonstrate that the iTRAQ-based quantitative mass spectrometry technique can reproducibly detect protein expression levels across samples in our analysis.

##### Incongruity Between mRNA and Protein Variation

To evaluate the concordance between mRNA and protein variation, RNA-seq data were collected from 84 inbred lines using the same tissues as proteomic analysis (see Experimental Procedures for details). A total of 2678 genes with the reliable measurements for both mRNA and protein abundance were used in the comparison (supplemental Table S3). We found that 66.7% of genes showed positive correlations between mRNA and protein abundances, whereas only 5.3% were statistically significant (FDR threshold of 0.05) (supplemental Fig. S2*A*). Next, we further conducted correlation analysis using the set of genes with significant variation (top 50% MAD value). Consistently, we observed a low degree of positive correlation between protein and mRNA levels (supplemental Fig. S2*B* and S2*C*), indicating that variation in protein levels is not entirely regulated through RNA expression as mentioned earlier in yeast, mouse and human studies (32–35, 77).

The low correlation between transcript and protein may simply reflect the prospect for post-transcriptional regulation (*e.g.* translation efficiency or protein modification including degradation). Alternatively, the global correlation may be convoluted by the superimposed combinations of a wide range of mRNA-protein correlations, which are assumed to vary in term of each individual gene or genes involved in the different biological pathway (54, 78–80). To test whether the orchestrated correlation between mRNA and protein variation could be indicated in distinct biological pathways, we performed the Kolmogorov-Smirnov test using the MapMan categories (supplemental Fig. S2*D*). The results showed that genes involved in the carbohydrate metabolism (CHO) pathways exhibited significantly positive correlation, whereas genes involved in protein metabolism displayed significantly low or negative correlations. Interestingly, the positive correlation of genes involved in metabolic process and the negative correlation of genes involved in protein metabolism have also been observed in previous mouse and human studies (54, 78–80). Taken together, these results suggest that mRNA measurement is a poor predictor of protein abundance variation in maize natural population, and biological functions of the gene products may affect the mRNA-protein correlation. Vice versa, posttranscriptional mechanisms likely play an important role in orchestrating the biological functions for genes involved in the same biological pathway.

##### Protein Coexpression Network

**Fig. 2.**
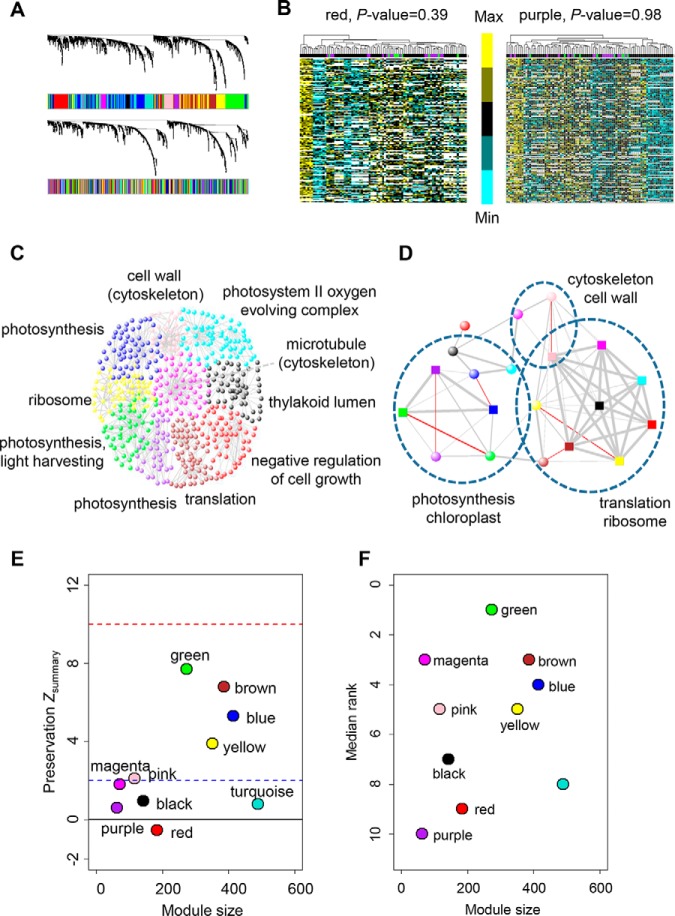
**Comparison of protein and mRNA networks.** *A*, WGCNA derived coexpression dendrograms and corresponding modules (colored boxes) for protein network (upper). We imposed the module definitions from protein onto the mRNA network (lower). *B*, Heat maps depicting normalized protein expression levels for all genes (rows) in all the samples (columns; purple labels are NSS lines, green are SS and red represents TST) for red (left) and purple (right) protein modules. *C*, The global structure of the protein WGCNA network using the top 100 gene-gene interactions in each module. Modules were colored based on the WGCNA module default name, and representative enriched GO categories were used for the annotation of each network. *D*, Module overlaps between protein and mRNA networks. Dots corresponded to modules from the protein (ball) and mRNA (rectangle) networks. The dots colors were assigned according to modules derived from WGCNA. Line widths were scaled based on the significance of overlap between corresponding modules. Red lines indicate significant mRNA-protein network preservation. Position of the dots and length of the lines were arbitrary to aid visualization. *E*, *Z*_summary_ statistics of module preservation. Each dot represents a module, labeled by WGCNA module default name. The dashed blue and red lines indicate the thresholds *Z*_summary_ = 2 and *Z*_summary_ = 10, respectively. *Z*_summary_ statistic (In general, *Z*_summary_ > 10 means highly preserved, *Z*_summary_ in between 2 and 10 is weak to moderate preservation, *Z*_summary_ < 2 indicates no preservation. *F*, Median rank of module preservation. Each dot represents a module, labeled by WGCNA module default name. In general, modules with lowest rank are highly preserved.

Genes belong to the same protein complex or in the same biological pathway are expected to be coexpressed because of a coordinated regulation of biological components and processes (81). To define if this is the case, we group similarly expressed genes into modules to generate protein-based coexpression networks using the weighted gene coexpression network analysis (WGCNA) (41). Meanwhile, to control the subpopulation effects, a linear regression model was used for population stratification adjustment (see Experimental Procedures for details). Eventually, a total of 10 modules were identified (Fig. 2*A*, supplemental Fig. S3). We then imposed a threshold based on module membership (MM) values (R > 0.4) to make final module assignments. Using this method as previously reported (46), each module contained an exact number of assigned genes, and many genes were assigned to multiple modules, albeit with different strengths. Standard heat maps of protein expression for each module indicated that the subgroup confounder did not significantly impact the results (Fig. 2*B*, supplemental Fig. S4, Kruskal-Wallis test, *p* > 0.05). We then performed Gene Ontology (GO) enrichment analysis toward each module (48, 82) and found that all the modules could be enriched for at least one GO term (supplemental Table S4; FuncAssociate, permutation-based adjusted *p* < 0.05). To visualize module network, we uploaded the 100 connections with highest TOs (topological overlap) within the module into the VisANT program (Fig. 2*C*) (83). These results suggest that the genes involved in the same biological processes could be coordinately regulated at the protein level.

We also generated the mRNA-based coexpression networks using the same method mentioned above. An easy way to assess the global comparability of mRNA and protein networks is to correlate overall connectivity between the two data sets (46). The higher the correlations of the property show, the greater similarities between the two data sets will be called (46). Here we found a very weak preservation of the connectivity between protein and RNA networks (supplemental Fig. S5). To directly assess coexpression preservation between mRNA and protein on a module-by-module basis, mRNA modules were assigned colors as indicated in protein networks (Fig. 2*A*). In supplemental Fig. S6, MM values between protein and mRNA networks showed modest correlation, indicating weak module preservation. Based on the MM threshold (R > 0.4), we then observed a modest degree of protein-mRNA module preservation, and modules with significant overlap tend to have similar functional characterizations (Fig. 2*D*, Table I). These results indicate that although some modules are overlapped between mRNA and protein networks, there are many modules specific for protein networks (Fig. 2*D*, Table I).

**Table I:** Characterization and preservation of protein and mRNA network modules.

Protein module	mRNA module	Overlap number	Overlap *p* value	Preservation *Z*-score
p-black	m-black	77 (77)	1	0.95
p-blue	m-blue	111 (69)	7.32 × 10^−6^	5.3
p-brown	m-brown	126 (84)	5.93 × 10^−6^	6.8
p-green	m-green	120 (60)	1.13 × 10^−14^	7.7
p-magenta	m-magenta	77(55)	0.12	1.8
p-pink	m-pink	70 (41)	2.55 × 10^−4^	2.1
p-purple	m-purple	45 (22)	2.96 × 10^−4^	0.6
p-red	m-red	27 (33)	1	−0.54
p-turquoise	m-turquoise	108 (108)	1	0.8
p-yellow	m-yellow	117 (68)	3.04 × 10^−8^	3.9

For each protein network, significance of overlap with the corresponding mRNA module was presented in column 3–4. The expected number of overlapping genes was presented in parentheses. *p* values were adjusted for multiple comparisons. Column 5 measured module preservation.

In order to quantify the preservation of protein modules in mRNA samples more objectively, one needs to consider statistics that does not rely on a module assignment in the mRNA data (47). Rigorous module preservation analysis based on *Z*-summary statistic results (see Experimental Procedures for details) showed that half of the modules exhibited weak to moderate preservation (2 < *Z*-score < 10), whereas all other modules exhibited little preservation (*Z*-score < 2) in the mRNA data (Fig. 2*E*, Table I). It is known that the *Z*-summary statistics tends to increase with module size (46, 47). Therefore, to measure relative preservation regardless of module size, a rank-based statistic median-Rank was proposed. A module with lower median rank trends to be more preserved than a module with a higher median rank (47). Combining the two preservation statistics, 4 of 10 modules (green, brown, blue and yellow) were found to be relatively stable because of their *Z*-summary statistics (*Z*-score > 2) (Fig. 2*E*) and median rank statistics close to minimum (Fig. 2*F*). Taken together, these results indicate that mRNA- and protein-based coexpression networks are relatively independent of each other.

##### Proteomic Subtypes of Maize Inbred Lines

It has been well-demonstrated in the previous studies that the genetic relationship among maize inbred lines would be readily recognized by their genomic relationships (84–86). However, nothing is known about the extent of such a genetic relationship when manifested at the proteome level. To address this issue, the most variable proteins and mRNAs (top 50%) among all the samples were subject to consensus clustering analysis, a method well-established for the reliable identification of proteomic and transcriptomic subtypes (see Experimental Procedures for details) (51, 52). Based on both visual inspection of the consensus matrix (supplemental Fig. S7*A*) and the delta plot assessing change in consensus cumulative distribution function (CDF) area (supplemental Fig. S7*B*), four segregated subgroups were observed for proteomic cohort (supplemental Fig. S7*C*). It is noted that because it's difficult to interpret the biological meanings of the small clusters, we removed the subgroups with less than 2 samples. Therefore, two remaining major subgroups, designated as subtype PA (*n* = 56) and subtype PB (*n* = 26), were considered in the following analysis (supplemental Fig. S7*D*). Likewise, after removing 7 samples in 3 small clusters, the transcriptomic cohort (*n* = 73) was also divided into two major subgroups (supplemental Fig. S8), designated as subtype TA (*n* = 49) and subtype TB (*n* = 17).

**Fig. 3.**
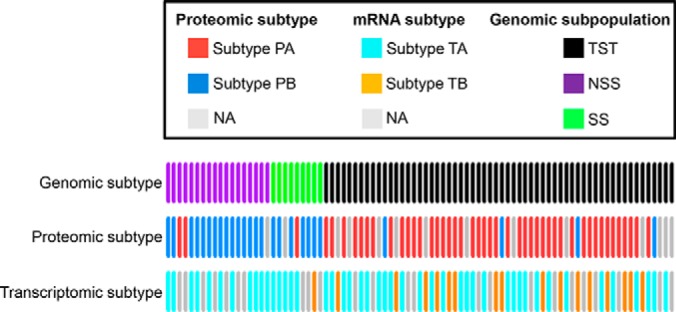
**Association of proteomic or transcriptomic subtypes with genomic subpopulation.** The gray lines indicate the missing lines in proteomic or transcriptomic subtype analysis.

Silhouette analysis (55) demonstrated that 73 of 82 samples in the proteomic dataset had positive silhouette width (supplemental Fig. S7*D*), indicative of a higher similarity to their own class than any other class members. These samples were hereby called as “core samples” and representative of their cluster assignments (56). The remaining 9 samples with negative silhouette width were removed from subsequent analyses. Likewise, 63 of 66 samples in the transcriptomic data sets were identified as core samples (supplemental Fig. S8*D*). Lastly, the lines with the close association were compiled together and compared with genomic subpopulation using Fisher's exact test (Fig. 3).We found that almost all the TST individuals were included in subtype PA and most of NSS/SS individuals were included in subtype PB (Fig. 3). In addition, we identified that the grouping of subtype PA and PB was dependent on the genomic division (Table II, *p* < 0.05). These results indicate that the proteome subtype could resemble the genomic subpopulation. However, there was less significant pattern in transcriptomic subtypes regarding genomic subpopulation (Fig. 3; Table II).

**Table II:** Association of proteomic and mRNA subtypes with genomic features.

Fisher's exact test	Subtype PA	Subtype PB	Subtype TA	Subtype TB
NSS	4.87 × 10^−7^*	4.87 × 10^−7^^$^	5.55 × 10^−2^	5.55 × 10^−2^
SS	2.42 × 10^−3^*	2.42 × 10^−4^^$^	1.86 × 10^−1^	1.86 × 10^−1^
TST	1.06 × 10^−11^^$^	1.06 × 10^−11^*	5.60 × 10^−3^	5.60 × 10^−3^
Temperate	1.06 × 10^−11^*	1.06 × 10^−11^^$^	5.60 × 10^−3^	5.60 × 10^−3^

^$^ indicates significantly more events in one cluster compared to the other one (*p* < 0.05);

* indicates significantly less events in one cluster compared to the other one (*p* < 0.05).

To further prove the finding that proteome could resemble better than transcriptome to the genomic subpopulation, we performed principal component analysis (PCA) for the samples based on proteome and transcriptome, respectively. The result showed that inputting proteome can present a clearer temperate-tropical division than transcriptome (supplemental Fig. S9*A* and S9*B*). In addition, we plotted the correlations of samples based on proteome or transcriptome with the genetic distance based on SNPs using TASSEL 5.2 (69). We found that using proteome yielded a more significantly negative correlation with genetic distance than with transcriptome (supplemental Fig. S9*C* and S9*D*). Taken together, these results suggested that the maize proteome may evolve under greater evolutionary constraint than transcriptome.

To better understand the biological implication underlying the proteomic subtypes, we identified protein signatures associated with different subtypes by supervised comparison of protein abundance in one subtype against the other one. As listed in supplemental Table S5, a total of 549 proteins exhibited significantly different abundances between the two proteomic subtypes (two-sided Wilcoxon rank-sum test, Bonferroni adjusted *p* < 0.05). A low overall cross-validation error rate of 2.7% supported a good generalizability of the subtypes and their signature proteins (see Experimental Procedures for details). Then GO enrichment analysis was performed to identify the subtype signatures. We found that proteins involved in the response to stress were significantly enriched in the down-signature of subtype PA (supplemental Fig. S10, FuncAssociate, permutation-based adjusted *p* < 0.05). In contrast, proteins related to chloroplast compartment or photosynthesis were highly enriched in the up-signature of subtype PA (supplemental Fig. S10, FuncAssociate, permutation-based adjusted *p* < 0.05).

##### Integrative Analysis of Protein Levels, RNA Expression and Genomic Subpopulation

Although the correlation analysis had revealed that the abundance of protein and mRNA was less coordinated, the interdependencies of such coordination in the context of distinct genomic subpopulation are not captured. To search genes capable to distinguish the genomic subpopulation when considering two levels of gene expression, we performed the self-organizing maps (SOM) analysis, an integrative machine learning method to project the high-dimensional typology of the relationships simultaneously (see Experimental Procedures for details) (61). To avoid the potential skewness of distance calculation because of difference in scale and variance of the input variables, we first converted each measurement into its relative rank order and designated it as percentiles to ensure the equal weighting of the input variables prior to the SOM training (see Experimental Procedures for details) (26). After training, each neuron within the SOM contains genes sharing a similar pattern of gene expression in the three genomic subpopulations (supplemental Fig. S11).

**Fig. 4.**
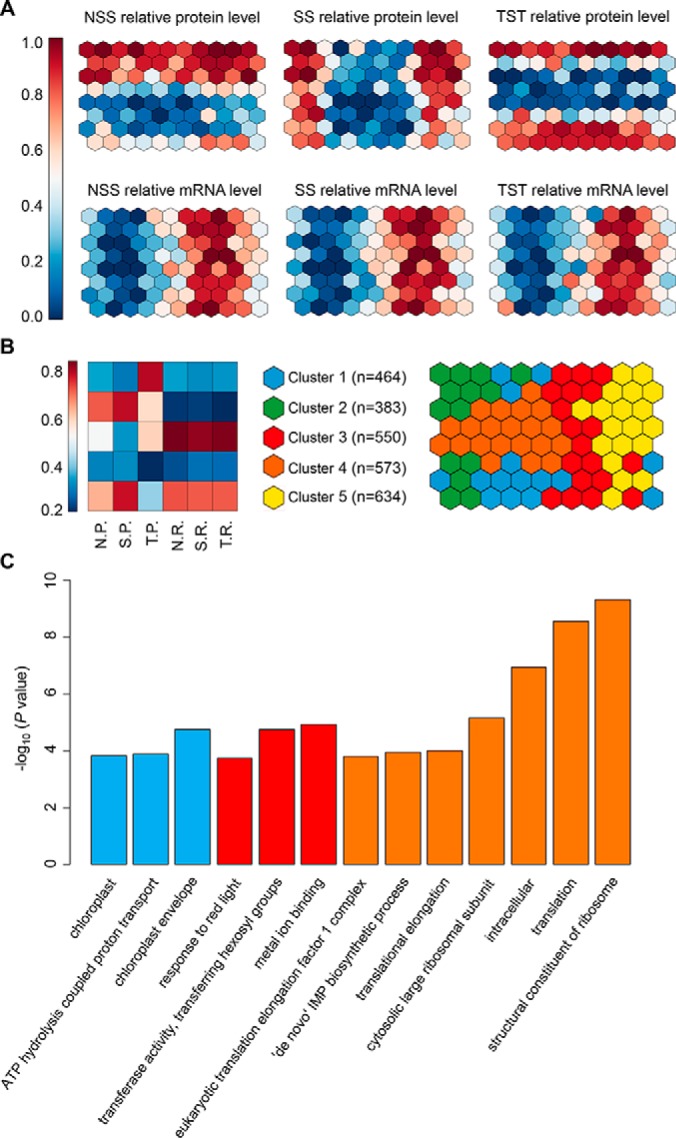
**The interdependencies between protein levels, RNA expression and genomic subpopulation.** *A*, Six different colorings of the trained SOM illustrated the relative mean levels of protein and mRNA in the three subpopulations for each neuron. *B*, Neurons were grouped using affinity propagation clustering (65). Shared coloring between nodes specifies membership to the same cluster. For each cluster, the mean rank of protein levels and mRNA levels in NSS, SS and TST subpopulations was shown for the representative neuron of the cluster. *C*, In three out of five clusters, significantly enriched gene ontology (GO) terms were identified (permutation-based corrected *p* < 0.05) (48). The bars were colored according to the cluster colors shown in Fig. 4*B*.

The rising map recapitulated the pairwise relationships between protein levels, RNA expression and genomic subpopulation across neurons (Fig. 4*A*). We further grouped neurons in the SOM using an affinity propagation clustering (65), to uncover five clusters in the SOM (Fig. 4*B*). Interestingly, the levels of mRNA expression did not show any obvious differences among genomic subpopulations in the same cluster (Fig. 4*B*). In contrast, we found distinct patterns of protein levels between different genomic subpopulations in some clusters (Fig. 4*B*). In detail, genes in cluster 5 have relatively higher protein expression in NSS and SS than that in TST subpopulation (Fig. 4*B*). In contrast, genes in cluster 1 have relatively lower protein expression in NSS and SS than that in TST subpopulation (Fig. 4*B*).

After examining the functional enrichments across each individual clusters within the SOM, we found that the enrichments in term of distinct biological pathways were present in three out of five clusters (Fig. 4*C*). For instance, genes encoding ribosome proteins or involved in translation process were enriched in Cluster 4 (Fig. 4*C*, FuncAssociate, permutation-based adjusted *p* < 0.05). In Cluster 1, genes with higher protein levels in TST than NSS and SS while mRNA maintaining stable, was significantly enriched for chloroplast compartment (Fig. 4*B* and *4C*, FuncAssociate, permutation-based adjusted *p* < 0.05). These findings suggest that maize may adapt to changes from tropical to temperate regions by deliberately modulating the different levels of mRNA and protein for some genes involving in specific biological processes.

##### Genetic Determinants of Variability in Protein Abundance

We next investigated whether genetic differences between individuals were associated with the observed variation in gene expression at the protein level. Considering the population structure and genetic relatedness among the inbred lines (67, 76), we used the mixed linear model (MLM) for association analysis toward a total of 2,657 genes. At a FDR threshold of 5% (corresponding to *p* < 1.98 × 10^−4^), we identified 281 *cis*-pQTLs distributed throughout 267 genes (here defined as those within 200 kb either side of the corresponding gene) (Table III and supplemental Table S6) and 29 *trans*-pQTLs (supplemental Table S7).

**Table III:** Number of cis-QTLs identified at the protein and mRNA levels.

Measurement	No. of lines	Significance	Cis-genes	*Cis*-QTLs
Protein levels	98	1.98 × 10^−4^	267	281
mRNA levels	84	3.47 × 10^−4^	434	461

**Fig. 5.**
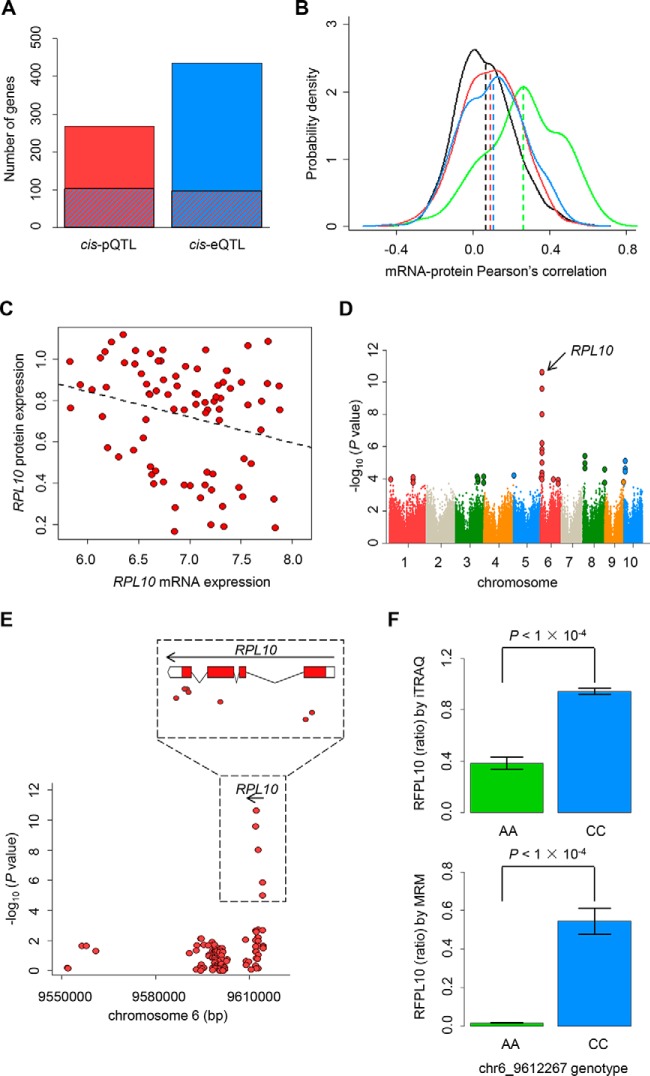
**Genetic loci associated with protein expression levels.** *A*, Overlap between genes with *cis*-pQTLs and *cis*-eQTLs. Red hues indicated 267 genes with *cis*-pQTLs, and the blue shading lines represented those genes simultaneously with *cis*-eQTLs. Blue hues indicated 434 genes with *cis*-eQTLs, and the red shading represented genes simultaneously with *cis*-pQTLs. *B*, Density plot of Pearson's correlations for each gene's protein and mRNA levels in four classes: with both pQTL and eQTL (green), with pQTL,but no eQTL (red), with eQTL, but no pQTL (blue) and no both pQTL and eQTL (black). *C*, Pearson's correlation between *RPL10* mRNA and protein expression levels. Protein and mRNA levels showed a poor correlation (*r* = −0.24, *p* = 0.02). *D*, Identification of *cis*-pQTLs for RPL10 protein. The *p* value and genomic coordinates for each protein/*cis*-SNP association test was plotted in the Manhattan plot. SNPs with significance threshold (Benjamini-Hochberg adjusted *p* < 0.05) were highlighted with a bigger dot size. The arrow indicates the location of the *RPL10* gene with a significant *cis*-pQTL. *E*, Overview of RPL10 protein level and SNP genotype association. The bottom plot was the fine mapping of *cis*-pQTL for the RPL10 protein. Each dot represents a tested SNP. The arrow depicts the chromosome location and transcription direction of the *RPL10* gene. There were several highly significant SNPs in the *RPL10* gene region. The exact locations of these SNPs in the *RPL10* gene region were illustrated in the top plot. The most significant SNP was chr6_9612267 and located at the fourth exon of *RPL10. F*, The bar plots showed the mean of RPL10 protein level of each chr6_9612267 genotype in 11 representative inbred lines, and the date were collected from iTRAQ (upper) and MRM (lower). Error bars denoted standard error of the mean.

To test what extent of the genetic determinants affecting protein levels coincides with those regulating mRNA levels, the genetic regions modulating mRNA expression (*cis*-eQTLs) were also identified and then compared with the pQTLs. A total of 461 *cis*-eQTLs were detected among 434 genes (FDR adjusted threshold of 5%, *p* < 3.47 × 10^−4^, Table III, supplemental Table S8), whereas none of *trans*-eQTLs were identified under the same threshold. Among 267 genes with *cis*-pQTL, 102 genes had at least one significant *cis*-eQTLs (Fig. 5*A*, 5% FDR adjusted threshold, *p* < 1.07 × 10^−3^). Vice versa, the significant *cis*-pQTL was present in 96 out of 434 genes with *cis*-eQTL (Fig. 5*A*, 5% FDR adjusted threshold, *p* < 4.25 × 10^−4^). In addition, after treating mRNA levels and population structure as covariates (conditional model, see Experimental Procedures for details), we identified a total of 176 protein-specific *cis*-QTLs (LRT, likelihood ratio test, Bonferroni adjusted *p* < 0.05, supplemental Table S9). Moreover, we found that genes with concordant QTL generally showed higher correlations between protein level and mRNA abundance than those with only *cis*-pQTL, *cis*-eQTL or neither (Fig. 5*B*).

To investigate the unique genomic features associated with each QTL, we used a hypergeometric test to compare the relative proportions of *cis*-QTLs *versus* all *cis*-SNP within the *cis*-genes identified (Table IV). SNPs within transcribed regions [exon and in the untranslated region (UTR)] are more highly enriched in both *cis*-pQTL and *cis*-eQTL than intron or sites located out of the genes tested (Table IV). In addition, the extent of *cis*-eQTLs enrichment for intron was much higher than *cis*-pQTLs, suggesting the functional role of intron transcription or splicing in affecting mRNA expression (Table IV). Taken together, these results have shown that although a substantial fraction of regulatory component can influence gene expression at both levels of mRNA and protein abundances, there are also several specific effects on particular expression phenotype.

**Table IV:** Enrichment of genomic annotations among pQTLs and eQTLs.

Annotation	pQTL	Background	*p* value	eQTL	Background	*p* value
CDS	91	3,043	8.77 × 10^−43^	237	4,674	1.24 × 10^−167^
5′UTR	11	464	5.09 × 10^−5^	51	890	3.29 × 10^−35^
3′UTR	33	1,666	1.62 × 10^−10^	85	2,664	4.96 × 10^−39^
Intronic	8	617	1.95 × 10^−2^	33	767	2.49 × 10^−19^
Extragenic	138	46,359	1	330	74,142	1

Manual inspection of the individual *cis*-pQTLs revealed many interesting variants in several cases. *RPL10* (ribosomal protein L10, *Zm00001d035201*) encodes a putatively structural constituent of ribosome protein. There was a poor correlation between *RPL10* protein and mRNA level (*r* = −0.24, *p* = 0.02, Fig. 5*C*), and we could only identify a *cis*-pQTL for *RPL10* (Fig. 5*D*). The variant exhibiting the strongest correlation with *RPL10* protein level is located at the fourth exon of the *RPL10* gene (chr6_9612267, *p* = 2.23 × 10^−11^, Fig. 5*E*). We validated this SNP by performing targeted proteomics with multiple reaction monitoring (MRM) analysis, and the result confirmed that the C allele was associated with higher protein level than the A allele as revealed by the iTRAQ data (supplemental Table S10, Fig. 5*F*). Therefore, this pQTL may affect protein abundance independent of the transcription, consistent with a post-transcriptional mode of regulation.

A second example, *NFD2* (nuclear fusion defective 2, *Zm00001d012824*) encodes a putative Ribonuclease III family protein involved in RNA processing (87). *NFD2* protein and mRNA levels were highly correlated (*r* = 0.49, *p* = 2.81 × 10^−5^, supplemental Fig. S12*A*). As expected, we found both *cis*-pQTL and *cis*-eQTL for *NFD2* (supplemental Fig. S12*B*). The most significant SNPs for *NFD2* protein and mRNA (chr5_929537, *p* = 1.34 × 10^−7^; chr5_929498, *p* = 7.51 × 10^−7^, respectively) located at 3′ downstream of the *NFD2* gene (supplemental Fig. S12*C*). We also validated the SNP (chr5_929537) by conducting MRM, and the results confirmed that the inbred lines with A allele tend to have higher protein levels than T allele (supplemental Table S10, supplemental Fig. S12*D*). These results suggest that the SNP (chr5_929537) may affect NFD2 protein level via at least partially mediating the transcription.

## DISCUSSION

The wide spread of maize from tropical to temperate regions requires the periodical but deliberate process of adaption to cope with the changing environments, such as photoperiod, daily temperature, and disease susceptibility (7–9). The multifaceted signatures of adaptions on the maize genome have begun to draw attention. It is intriguing to recognize that the footprints of selection within the genome driving maize adaption occurred within a short evolutionary time frame, and the genetic split between TST and NSS/SS seems to be ∼3000 to 5000 years ago (10, 86). In addition, hundreds of genomic regions were shown to be selected and the candidate genes within the selected regions were enriched for stress response, developmental and metabolic processes (10). Moreover, a recent study has identified that the directional selection resulted in ∼14.4% of the total genes differentially expressed between tropical and temperate lines, indicating that the alterations in transcriptome may be prevalent during maize adaption (10).

It is well-known that the levels of transcripts could not entirely reflect the abundances of the corresponding proteins in cells because of many post-transcriptional events such as alternative splicing, translational efficiency, proper folding, transport and localization, assembly into complexes, and posttranslational modifications (21, 88). Early studies in yeast, mouse and human have revealed the importance of these post-transcriptional processes in shaping the accordance of transcript and protein level during inter- or intra-species evolution (22, 23, 89–91). In the present study, the matched proteomic and RNA-seq measurements for ca. 3,000 genes enabled the first global analysis of mRNA-to-protein correlation in a large maize association cohort. Although a limited mRNA-to-protein correlation was observed for individual genes across 84 inbred lines, we found that the CHO metabolic pathway was enriched for genes with high and positive correlations compared with the protein synthesis enriched for genes exhibiting weaker correlations. These results are consistent with previous studies in human and mouse (54, 78–80), and support the notion that although many biological functions are primarily regulated by mRNA abundance, post-transcriptional mechanisms likely have an important role in synchronizing expression regulation toward genes in certain biological pathways. Meanwhile, conceivably corresponding to the insignificant correlation between mRNA and protein abundance, we observed little preservation between mRNA- and protein-based coexpression networks, suggesting two coexpression networks yield divergent predictions of gene relatedness. An important implication of these results cautions ongoing efforts aiming to identify genetic variants by determining mRNA and protein expression. Indeed, we found that despite a substantial overlap between eQTLs and pQTLs, numerous pQTLs were distinct from eQTLs, suggesting that diverse regulatory genetic mechanisms influence gene expression phenotypes at many different levels in a natural maize population.

The genetic relationships among maize inbred lines would be precisely manifested after assessing their genomic correlation (75, 76). To understand the readout of gene expression according to genomic variation, we reclassified the maize inbred lines using proteome and transcriptome profiles superimposed on the genomic data. Surprisingly, we found that proteome subtype could resemble the established genomic subpopulation very well, whereas transcriptome subtype showed less consistency than genomic subpopulation. This result suggests that there seemed to be some 'compressed' mechanisms to force proteome to amend in accordance with genome specification during maize adaption. Therefore, extensive post-transcriptional regulation must help to compensate for divergent mRNA expression to maintain protein abundances at evolutionary preferred levels. The similar “compressed” change mechanism in proteome compared with change in transcriptome has been preliminarily documented across a large evolutionary distance (*e.g.* across bacteria, yeast, worms, flies and human cells) (21–23). It has been suggested that the amplified diversity in mRNA abundances may be a mechanism to increase chances of survival under stress conditions, whereas 'compressed' protein expression levels are presumably optimized to fit proper cellular functions (22). Such a speculation appears to hold true even within an intra-species population, such as in maize observed in our study. In agreement with this concept, we observed the protein levels of genes involved in the response to abiotic stress are commonly higher in NSS/SS than TST samples. The result supports the possibility that higher protein stability or feedback mechanisms on translation efficiency would modulate the distinct levels of stress response in accommodating maize adaption. It is worth noting that proteins used in our analysis are of relatively high abundant because of technical limitations. It remains intriguing to see whether high conservation of protein levels would also be applicable for low abundance proteins in different maize lines or in other plant intraspecies.

In summary, we describe the first systematic interrogation of the genetic effects on the plant proteome using iTRAQ mass spectrometry. Our proteomic characterization of the maize inbred lines with proved genomic genotype illuminates the power of integrated multi-omics analysis. The results demonstrate that protein abundance cannot be reliably predicted from RNA-level measurements. Protein and mRNA were correlated at a low extent, like the suggestions from the earlier studies in human, mouse and yeast (90, 92, 93). In addition, we identified that the proteomics-based subtyping was more like the genomic subpopulation than the subtypes defined by transcript profile. This result suggests that abundance changes in transcriptome may be effectively neutral, either buffered or compensated at the proteome during the adaption of modern maize from tropical to temperate regions.

## DATA AVAILABILITY

All mass spectrometry raw data were deposited in iProX (http://www.iprox.org) with accession number IPX0001097001. The raw mRNA sequence data reported in this paper have been deposited in the Genome Sequence Archive (94) in BIG Data Center (95), Beijing Institute of Genomics (BIG), Chinese Academy of Sciences, under accession numbers CRA000334 that are publicly accessible at http://bigd.big.ac.cn/gsa.

## Supplementary Material

supplemental Table S1

## Footnotes

^1^ The abbreviations used are:

eQTL: mRNA expression levels
mQTL: metabolic quantitative expression levels
pQTL: protein levels
LOOCV: leave-one-out cross validation
SOM: self-organizing maps.
