# Supplementary material for: Characterization of Proteome Variation During Modern Maize Breeding
Source: Mol Cell Proteomics. 2018 Nov 8;18(2):263–76. doi: 10.1074/mcp.RA118.001021 (PMC6356080; doi:10.1074/mcp.RA118.001021)
Supplement: supplemental Table S1 [file RA118.001021_index.html]

Supplement to Characterization of proteome variation during modern maize breeding | Molecular & Cellular Proteomics

## Supplemental Data

- Supplemental\_Table\_and\_Figure\_Legends - This Supplemental Information file includes Supplemental Table 1-10 legends and Supplemental Figures 1-12.
- Supplemental Table 1 - Supplemental Table 1. MRM transitions results.
- Supplemental Table 2 - Supplemental Table 2. Relative abundance (inbred lines vs. B73) of 2,750 proteins in 98 maize inbred lines.
- Supplemental Table 3 - Supplemental Table 3. FPKM values of 2,678 mRNA in 84 inbred lines.
- Supplemental Table 4 - Supplemental Table 4. All 10 modules could be enriched for at least one GO term
- Supplemental Table 5 - Supplemental Table 5. Signature proteins for proteomic subtypes.
- Supplemental Table 6 - Supplemental Table 6. cis-pQTLs at significance threshold Benjamini-hochberg P = 0.05.
- Supplemental Table 7 - Supplemental Table 7. trans-pQTLs at significance threshold P = 8.12 &#x00D7; 10-8.
- Supplemental Table 8 - Supplemental Table 8. cis-eQTLs at significance threshold Benjamini-hochberg P = 0.05.
- Supplemental Table 9 - Supplemental Table 9. Protein-specific cis-QTLs at significance threshold Bonferroni P = 0.05.
- Supplemental Table 10 - Supplemental Table 10. MRM quantification results.
