# Supplementary material for: Characterization of Proteome Variation During Modern Maize Breeding
Source: Mol Cell Proteomics. 2018 Nov 8;18(2):263–76. doi: 10.1074/mcp.RA118.001021 (PMC6356080; doi:10.1074/mcp.RA118.001021)
Supplement: supplemental Table S1 [file 139955_1_supp_230613_phrrbc.pdf]

Supplemental Information

This Supplemental Information file includes Supplemental Table 1-10 and Supplemental Figures 1-12.

Supplemental Tables:

Supplemental Table 1. MRM transitions results. (XLSX 57kb)

Supplemental Table 2. Relative abundance (inbred lines vs. B73) of 2,750 proteins in 98 maize inbred lines. (XLSX 6,441kb)

Supplemental Table 3. FPKM values of 2,678 mRNA in 84 inbred lines. (XLSX 3,263kb)

Supplemental Table 4. All 10 modules could be enriched for at least one GO term (XLSX 20kb)

Supplemental Table 5. Signature proteins for proteomic subtypes. (XLSX 24kb)

Supplemental Table 6. *Cis*-pQTLs at significance threshold Benjamini-hochberg P = 0.05. (XLSX 32kb)

Supplemental Table 7. *Trans*-pQTLs at significance threshold $P = 8.12 \times 10^{-8}$ (XLSX 14kb).

Supplemental Table 8. *Cis*-eQTLs at significance threshold Benjamini-hochberg P = 0.05. (XLSX 39kb)

Supplemental Table 9. Protein-specific cis-QTLs at significance threshold Bonferroni P = 0.05 (XLSX 27kb)

Supplemental Table 10. MRM quantification results. (XLSX 11kb)

Supplemental Figure Legends:

Supplemental Fig. 1. Assessment of protein identification accuracy.

(*A*) The bimodality coefficient and dip *t*-test. If Hartigan's dip test $P > 0.05$ or bimodality coefficient < 0.555, the distribution was considered to be unimodal. Each point corresponded to a sample. (*B*) Distribution plot of the R^2 values for simple linear regression between pairs of non-

replicates with a mean correlation coefficient of 0.07 (black dashed line). We then calculated the R^2 values between the 7 replicate lines (mean value of 0.41, red dashed line). We carried out 100,000 permutations to judge whether the replicate lines R^2 values were signiicantly higher than non-replicates. ($P < 1 \times 10^{-5}$) . (*C*) The Spearman's correlation between the two biological replicates for the other six inbred lines.

Supplemental Fig. 2. Correlations between mRNA and protein abundance.

(*A*) mRNA and protein were correlated across all the 84 samples, resulting in 66.7% positive correlations, but only 5.3% of genes showed significant correlation (5% FDR using the Benjamini-Hochberg method), with a mean correlation coefficient of 0.081. (*B*) mRNA and protein were correlated using the set of genes with significant protein variation (top 50% MAD value), which resulted in 67.5% positive correlations, but only 8.9% being statistically significant (5% FDR using the Benjamini-Hochberg method). (*C*) mRNA and protein were correlated using the set of genes with significant RNA variation (top 50% MAD value), which resulted in 70.2% positive correlations, but only 9.7% being statistically significant. (*D*) mRNA and protein levels displayed different correlation for genes involved in different biological processes. *P* values from the Kolmogorov–Smirnov test were provided in the parentheses following the MapMan categories names.

Supplemental Fig. 3. Evaluation of soft thresholds used in WGCNA to create the Bicor based co-expression networks.

The power 9 was selected for protein co-expression network construction, which was the lowest power for which the scale-free topology fit index reaches 0.85. Scale independence of the protein co-expression network (A). Mean connectivity in the protein co-expression network (B).

Supplemental Fig. 4. Heat maps depicting normalized protein expression levels for all

genes (rows) in all samples (columns; purple labels are NSS lines, green are SS and red represents TST) for each module: yellow (A), blue (B), black (C), pink (D), magenta (E), turquoise (F), green (G), brown (H).

Supplemental Fig. 5. Gene connectivity showed less preservation between mRNA and protein. The x and y axes represented the rank of overall connectivity for protein and mRNA data sets, respectively. Dot plots (A) and density plots (B) presented the same information in different ways.

Supplemental Fig. 6. Module membership values (MM) correlation between mRNA and protein networks showing a weak to moderate preservation between the two networks. (A-J) Density plots illustrating MM comparison between the two networks for each module: yellow (A), red (B), blue (C), black (D), magenta (E), turquoise (F), green (G), purple (H), pink (I), brown (J).

Supplemental Fig. 7. Identification of proteomic subtypes and core samples

(*A*) Consensus matrixes of 87 maize inbred lines (11 lines in mixed subpopulation skipped) for each k (k=2 to 8), displaying the clustering stability using 1000 iterations of hierarchical clustering. (*B*) Plot of cumulative distribution function (CDF) from consensus matrix for each k (k= 2 to 8). (*C*) The Δ(k) vs k plot indicating the optimal cluster number of k=4 where the ‘elbow’ occurs. (*D*) Silhouette analysis identifying ‘‘core’’ samples defined as inbred lines with positive silhouette values.

Supplemental Fig. 8. Identification of transcriptomic subtypes and core samples.

(*A*) Consensus matrixes of 73 maize inbred lines (11 lines in mixed subpopulation skipped) for each k (k=2 to 8), displaying the clustering stability using 1000 iterations of hierarchical

clustering. (*B*) Plot of cumulative distribution function (CDF) from consensus matrix for each k (k= 2 to 10). (*C*) The Δ(k) vs k plot indicating the optimal cluster number of k=6 where the 'elbow' occurs. (*D*) Silhouette analysis identifying ''core'' samples defined as inbred lines with positive silhouette values.

Supplemental Fig. 9. Principal component analysis (PCA) results and smooth-scatter plot of sample-sample correlations based on protein or mRNA versus genetic distance.

(*A*) PCA analysis based on the protein data (top 50% MAD). (*B*) PCA analysis based on the mRNA data (top 50% MAD). Black triangle, purple circle and green rectangle indicate TST, NSS and SS, respectively. (*C*) Smooth-scatter plot of sample-sample correlations based on protein level versus genetic distance; (*D*) Smooth-scatter plot of sample-sample correlations based on mRNA level versus genetic distance.

Supplemental Fig. 10. Identification of the subtype signature proteins.

Supplemental Fig. 11. Workflow of the SOM training process.

A self-organizing map (SOM) was trained using relative protein and mRNA levels in NSS, SS and TST subpopulations. Prior to training, all of the measurements were converted into their relative rank order. Afterward, each neuron in the SOM represents genes sharing a similar pattern of gene expression among all three subpopulations.

Supplemental Fig. 12. Genetic loci associated with *NFD2* expression at two levels. **(*A*)** Pearson's correlation between *NFD2* mRNA and protein expression levels. There was a significantly positive correlation between mRNA and protein levels (r = 0.49, $P = 3.48 \times 10^{-5}$). **(*B*)** Identification of *cis*-QTLs for NFD2 expression at protein level (upper) and mRNA level (lower). The *P*-value and genomic coordinates for each gene/cis-SNP association test was plotted in the

Manhattan plot. SNPs with significance threshold (Benjamini–Hochberg adjusted $P < 0.05$) were highlighted with a bigger dot size. The arrow indicated the location of the NFD2 gene which contains a significant cis-QTL. **(*C*)** Overview of *NFD2* protein level and SNP genotype association. The bottom-plot was the fine mapping of *cis*-QTL for *NFD2* protein and mRNA. Each dot represented a tested SNP. The arrow depicted the chromosome location and transcription direction of the *NFD2* gene. The exact locations of the highly significant SNPs in the *NFD2* gene region were illustrated in the top plot. The most significant SNPs for both protein and mRNA levels located in 3’ downstream of *NFD2.* **(*D*)** The bar plots showed the mean of NFD2 protein level of each chr5_929537 genotype in 11 representative inbred lines, and the date were collected from iTRAQ (upper) and MRM (lower). Error bars denoted standard error of the mean.

Fig. S1

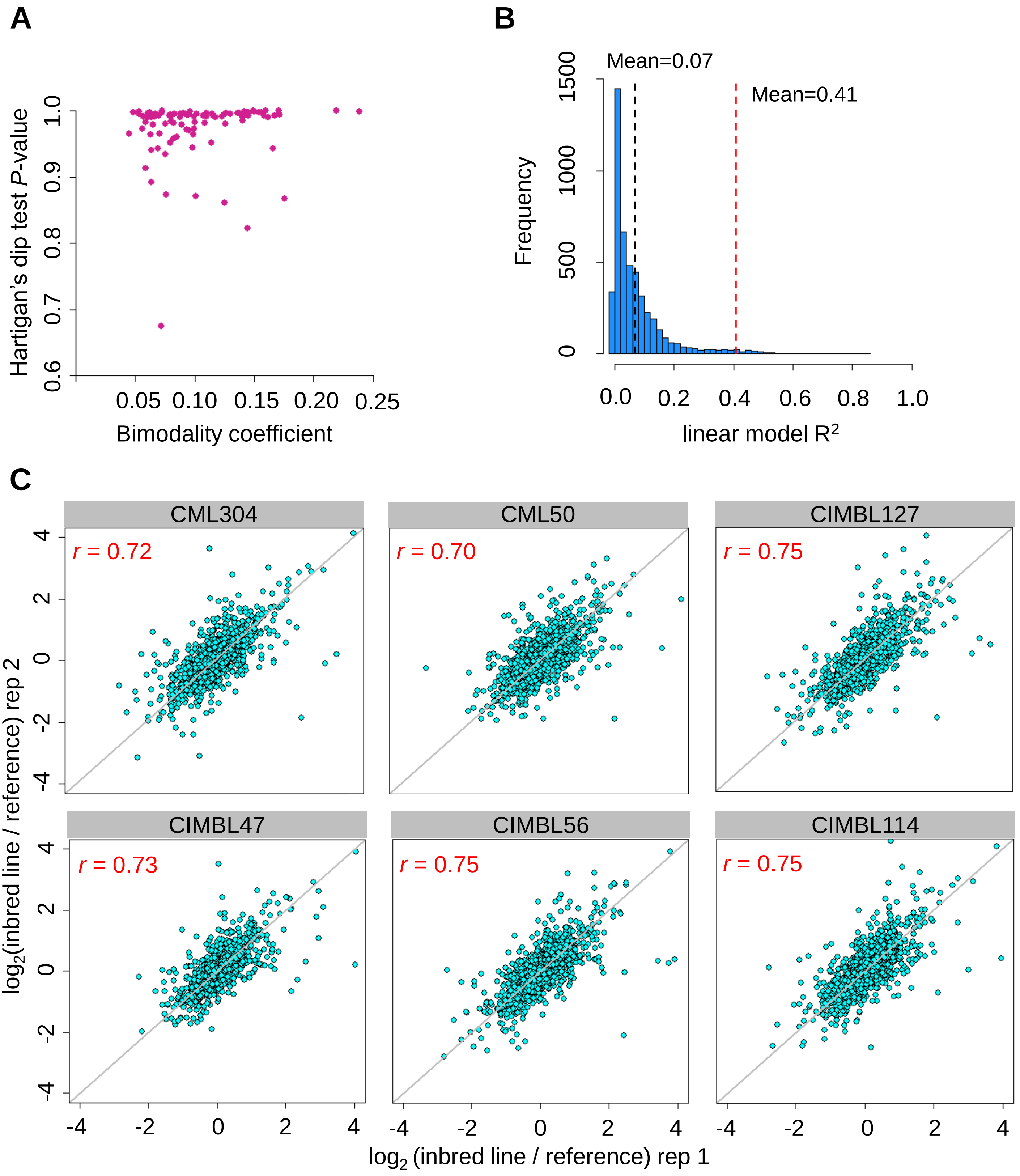

A
Hartigan's dip test P-value
Bimodality coefficient
B
Mean=0.07
Mean=0.41
Frequency
linear model R2
C
CML304
r = 0.72
CML50
r = 0.70
CIMBL127
r = 0.75
CIMBL47
r = 0.73
CIMBL56
r = 0.75
CIMBL114
r = 0.75
log2(inbred line / reference) rep 2
log2 (inbred line / reference) rep 1

Fig. S2

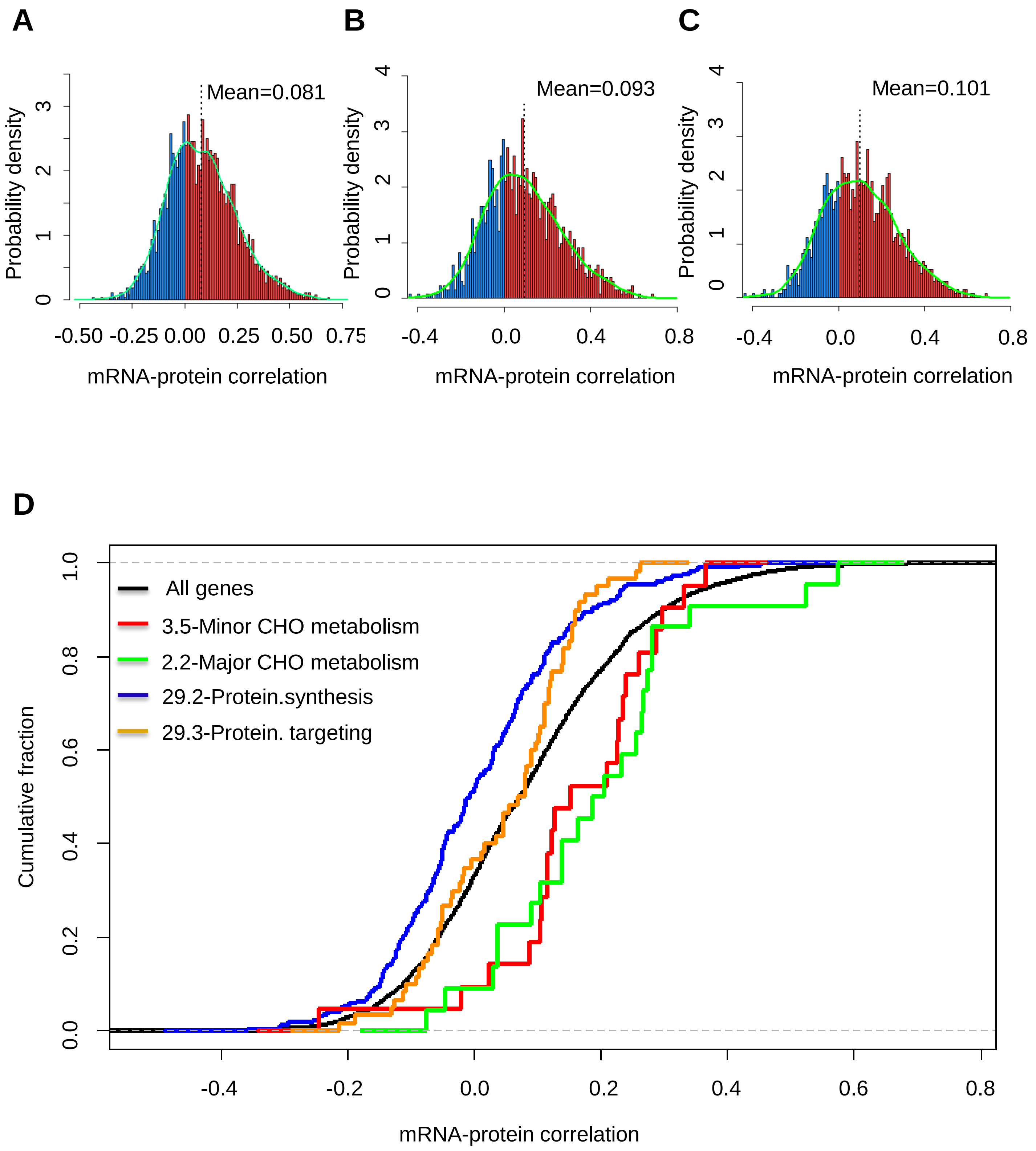
A
B
C
Mean=0.081
Mean=0.093
Mean=0.101
Probability density
mRNA-protein correlation
-0.50 -0.25 0.00 0.25 0.50 0.75
-0.4 0.0 0.4 0.8
D
Cumulative fraction
All genes
3.5-Minor CHO metabolism
2.2-Major CHO metabolism
29.2-Protein.synthesis
29.3-Protein. targeting
-0.4 -0.2 0.0 0.2 0.4 0.6 0.8
0.0 0.2 0.4 0.6 0.8 1.0

Fig. S3

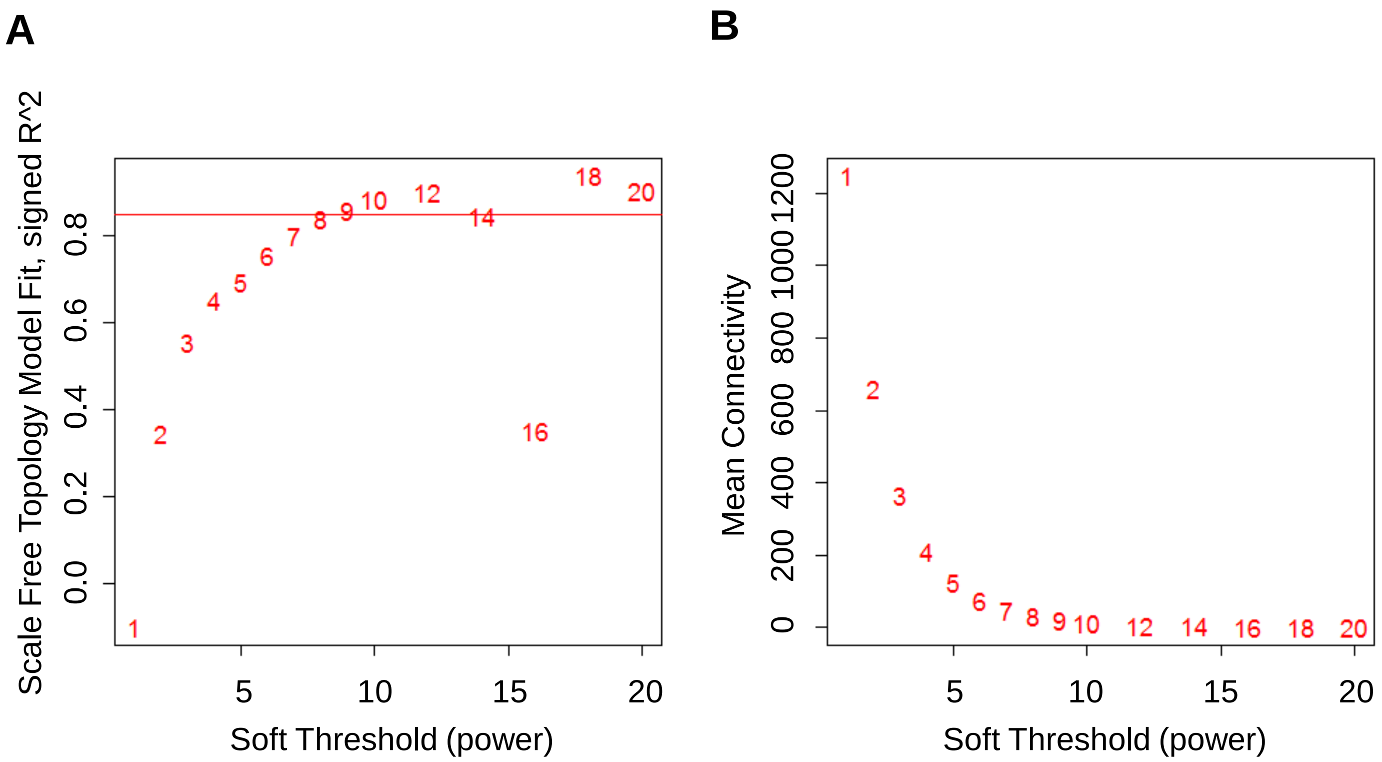
A
Scale Free Topology Model Fit, signed R^2
Soft Threshold (power)
B
Mean Connectivity
Soft Threshold (power)

Fig. S4

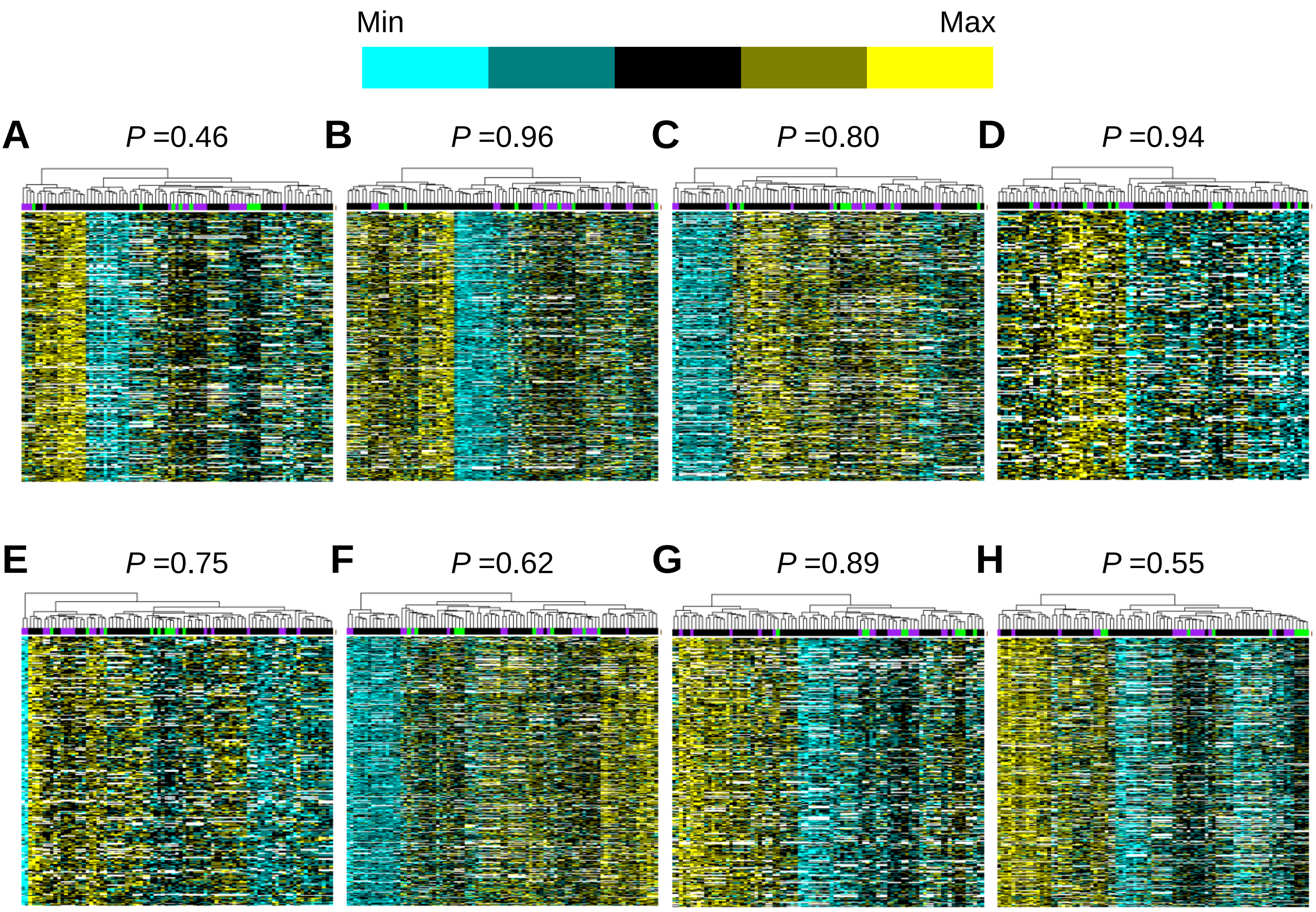

Fig. S5

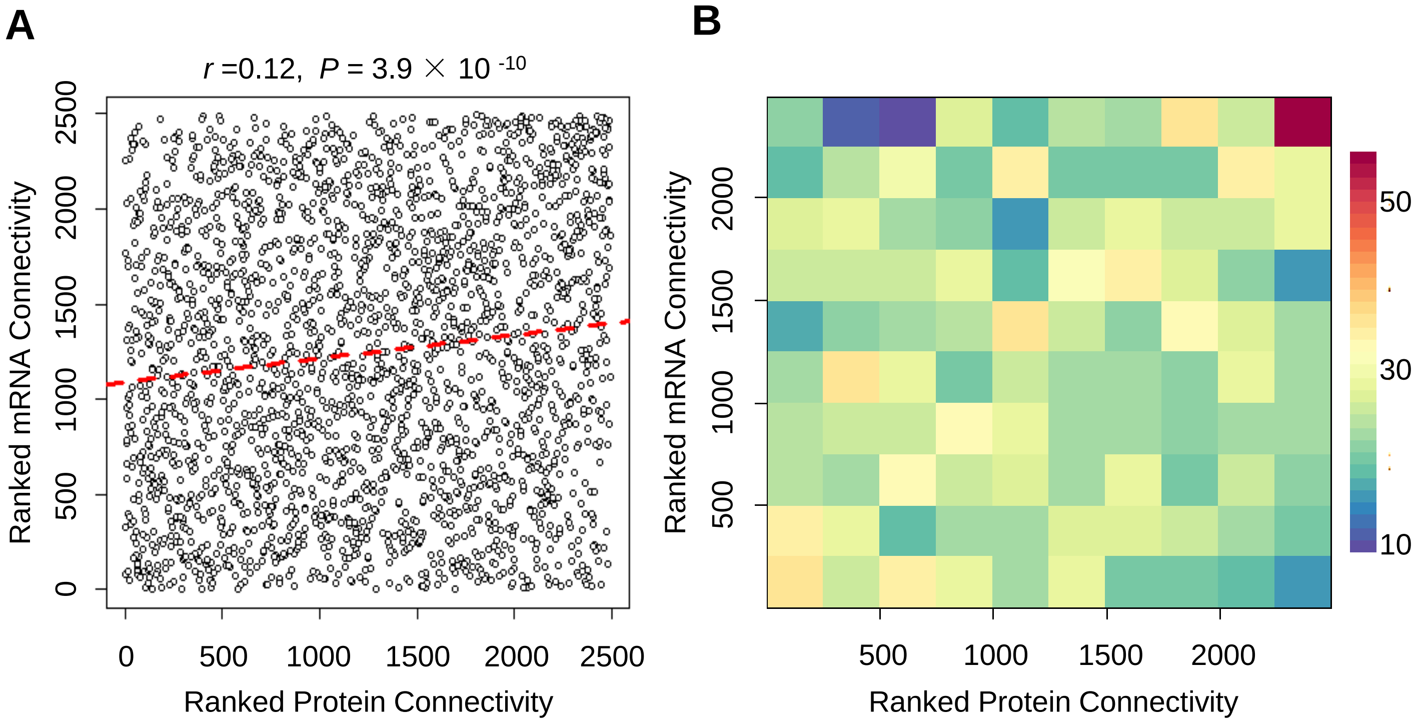
A
r =0.12, P = 3.9 × 10 -10
Ranked mRNA Connectivity
0
500
1000
1500
2000
2500
Ranked Protein Connectivity
B
Ranked mRNA Connectivity
500
1000
1500
2000
Ranked Protein Connectivity
50
30
10

Fig. S6

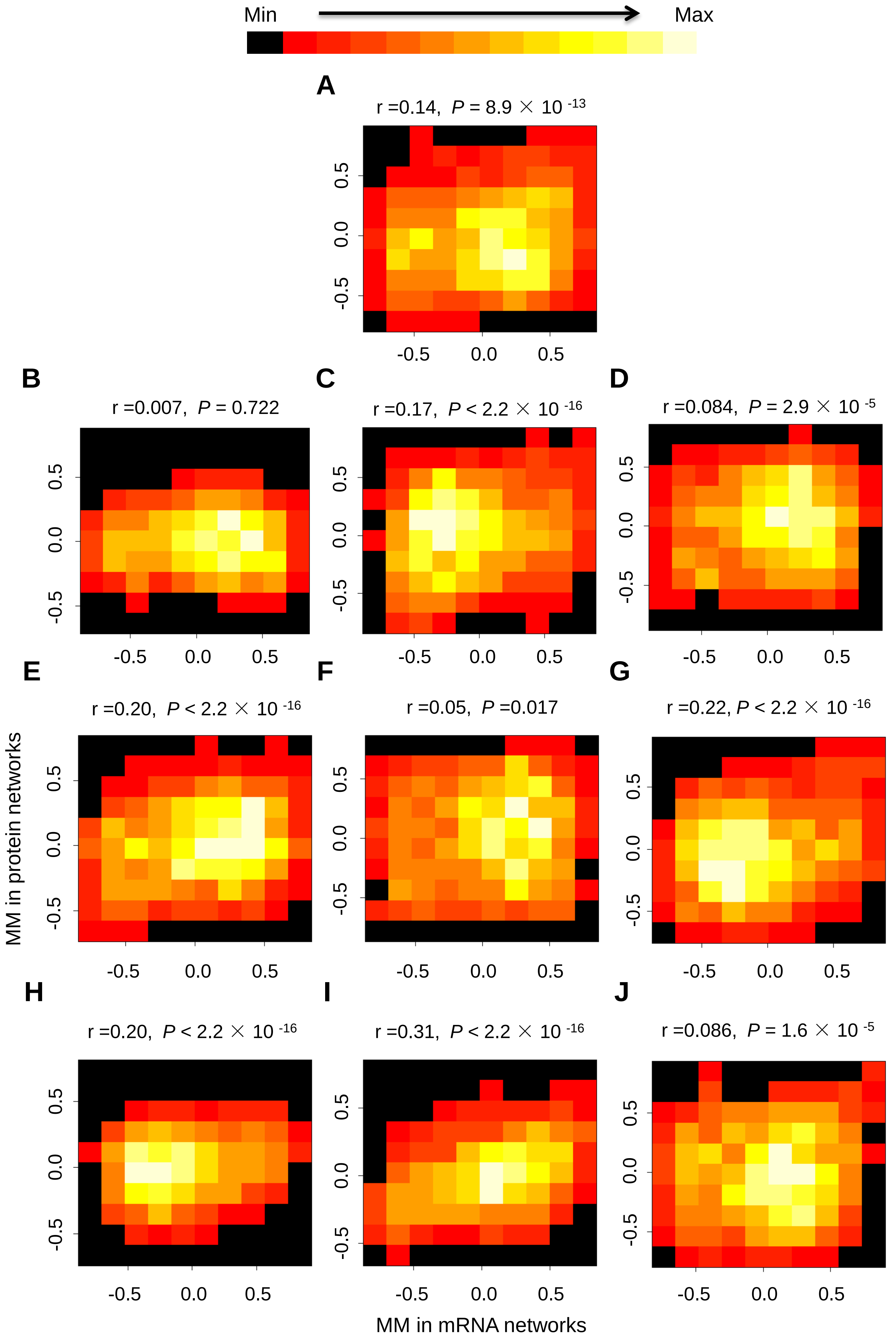

Min
Max
A
r =0.14, P = 8.9 × 10 -13
B
r =0.007, P = 0.722
C
r =0.17, P < 2.2 × 10 -16
D
r =0.084, P = 2.9 × 10 -5
E
r =0.20, P < 2.2 × 10 -16
F
r =0.05, P =0.017
G
r =0.22, P < 2.2 × 10 -16
H
r =0.20, P < 2.2 × 10 -16
I
r =0.31, P < 2.2 × 10 -16
J
r =0.086, P = 1.6 × 10 -5
-0.5
0.0
0.5
MM in protein networks
MM in mRNA networks

Fig. S7

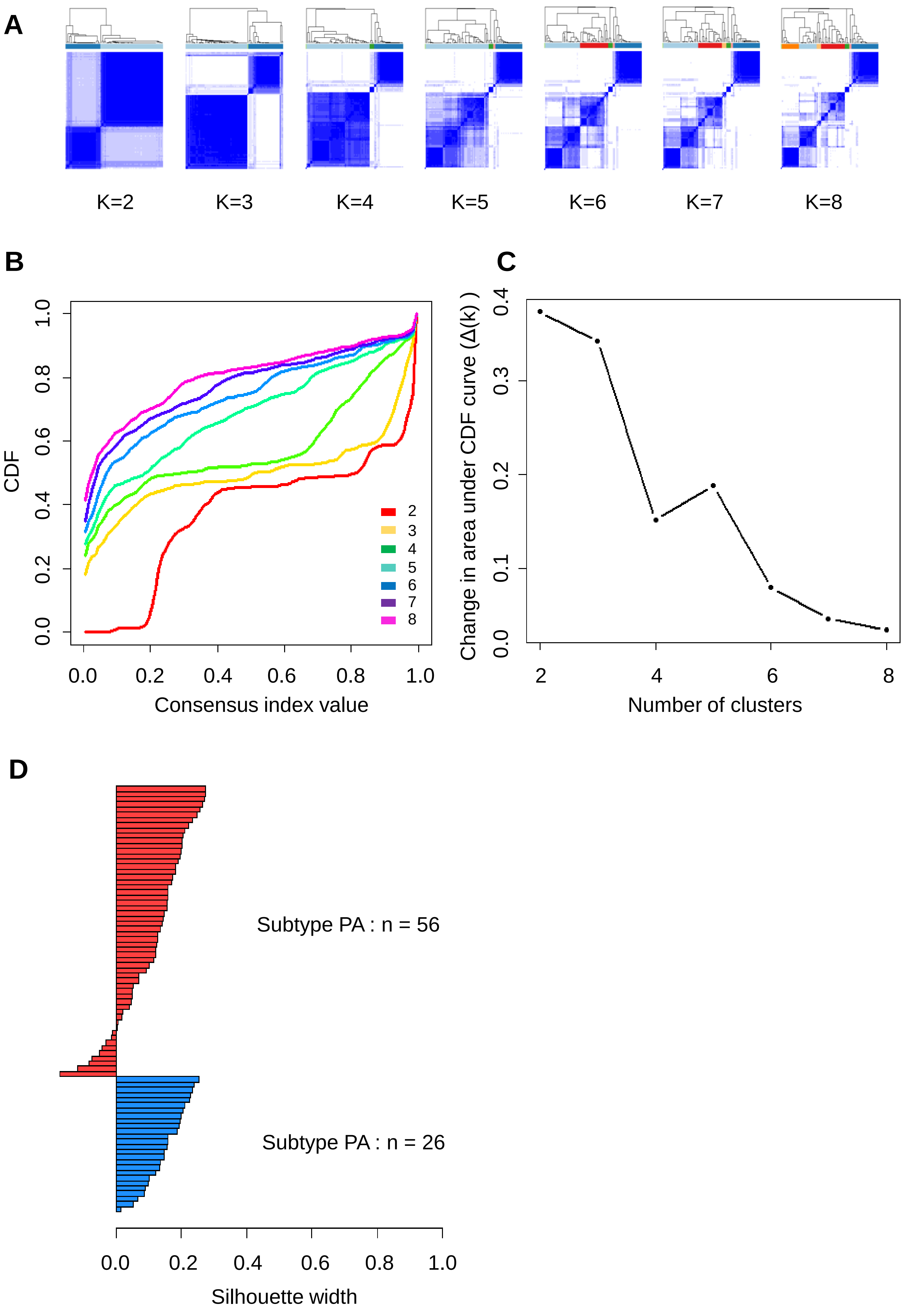
A
K=2
K=3
K=4
K=5
K=6
K=7
K=8
B
CDF
0.0
0.2
0.4
0.6
0.8
1.0
Consensus index value
2
3
4
5
6
7
8
C
Change in area under CDF curve (Δ(k))
0.0
0.1
0.2
0.3
0.4
2
4
6
8
Number of clusters
D
Subtype PA : n = 56
Subtype PA : n = 26
0.0
0.2
0.4
0.6
0.8
1.0
Silhouette width

Fig. S8

A

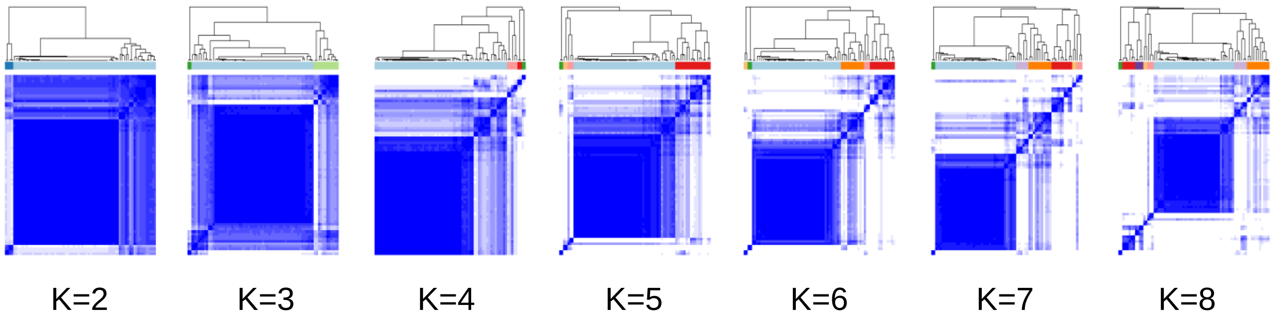

K=2
K=3
K=4
K=5
K=6
K=7
K=8

B

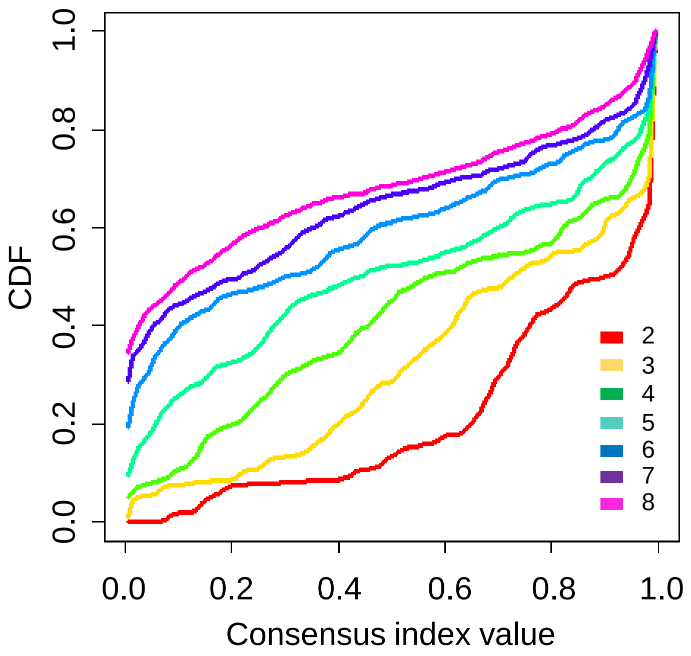

CDF
1.0
0.8
0.6
0.4
0.2
0.0
2
3
4
5
6
7
8
0.0
0.2
0.4
0.6
0.8
1.0
Consensus index value

C

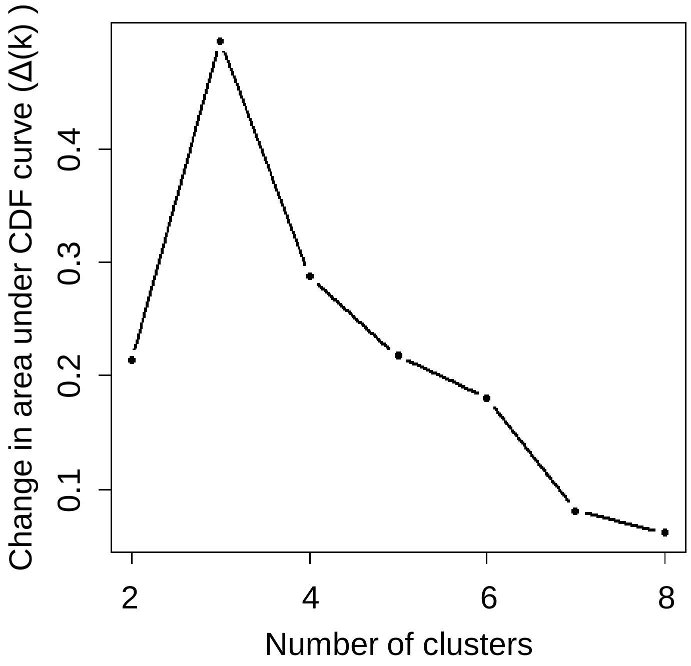

Change in area under CDF curve (Δ(k))
0.4
0.3
0.2
0.1
2
4
6
8
Number of clusters

D

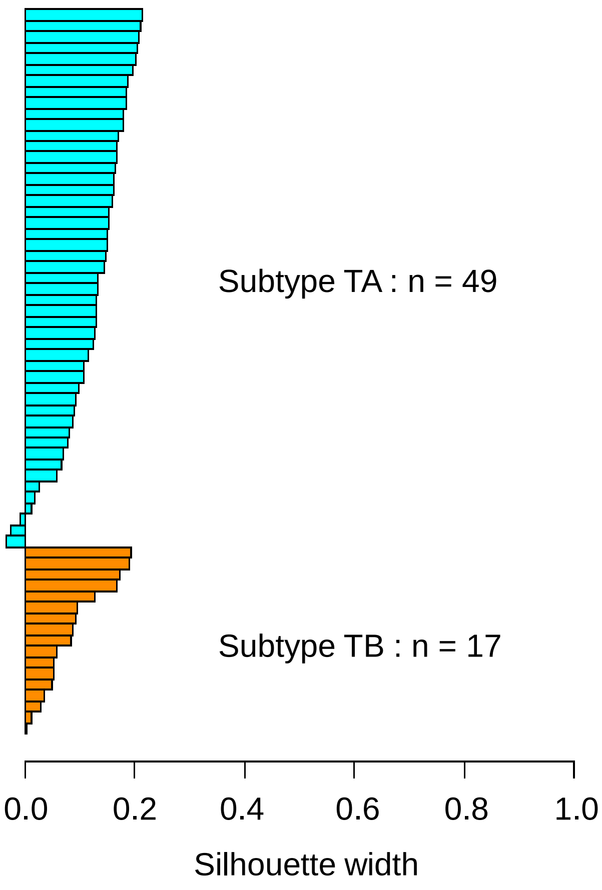

Subtype TA : n = 49
Subtype TB : n = 17
0.0
0.2
0.4
0.6
0.8
1.0
Silhouette width

Fig. S9

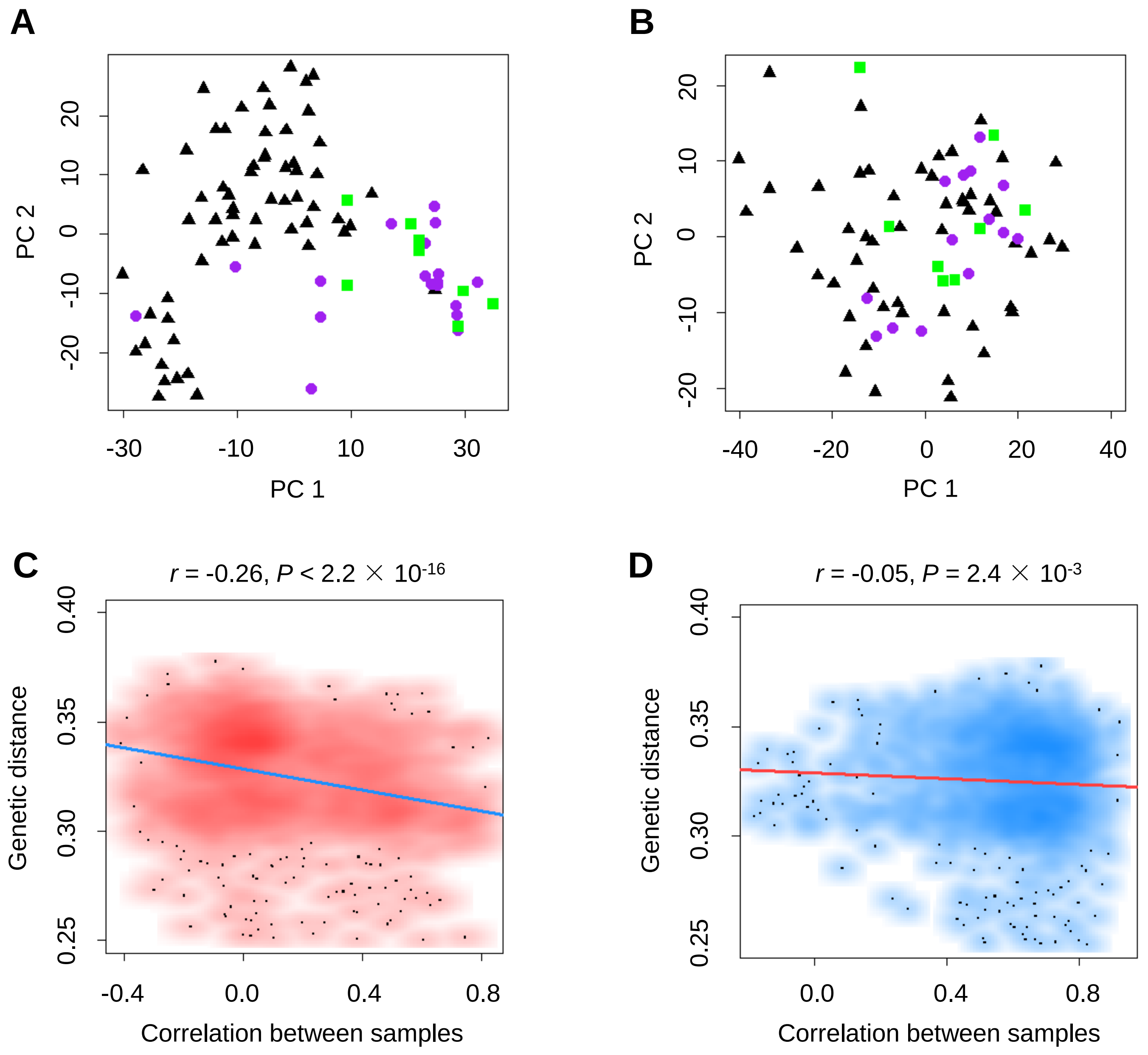
A
B
C
D
PC 1
PC 2
r = -0.26, P < 2.2 × 10-16
r = -0.05, P = 2.4 × 10-3
Genetic distance
Correlation between samples

Fig. S10

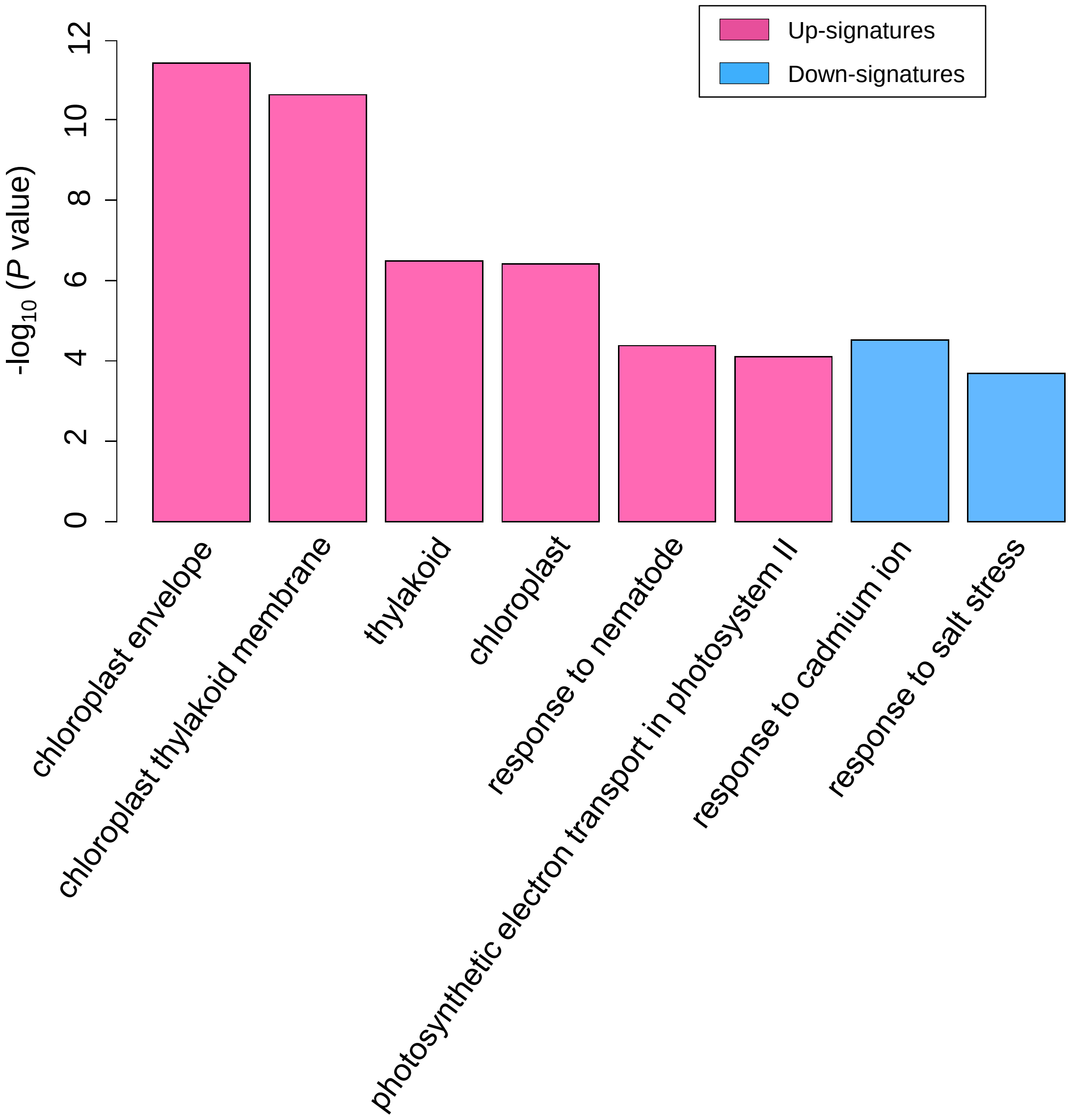
Up-signatures
Down-signatures
-log10 (P value)
0
2
4
6
8
10
12
chloroplast envelope
chloroplast thylakoid membrane
thylakoid
chloroplast
response to nematode
photosynthetic electron transport in photosystem II
response to cadmium ion
response to salt stress

Fig. S11

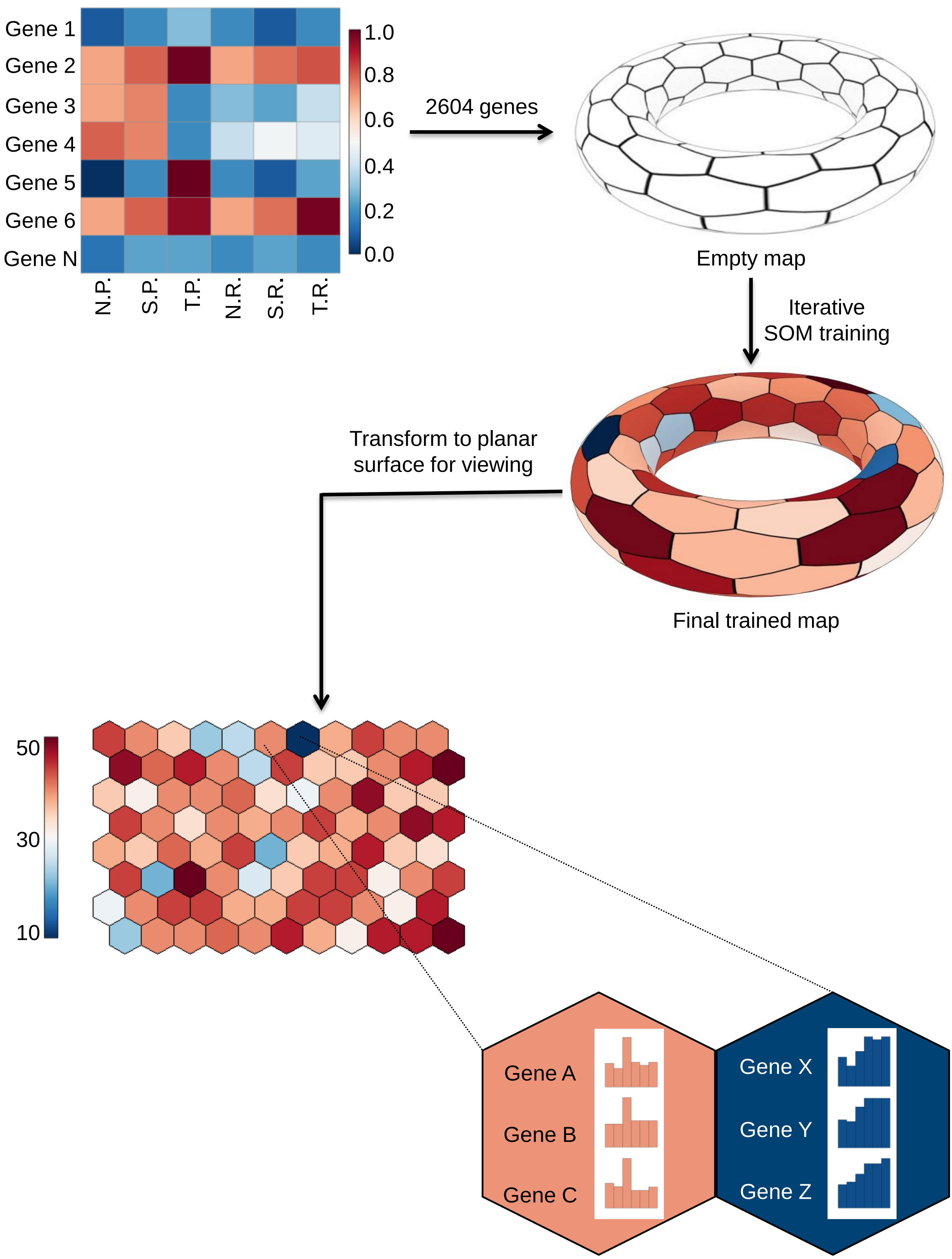

Gene 1
Gene 2
Gene 3
Gene 4
Gene 5
Gene 6
Gene N
N.P.
S.P.
T.P.
N.R.
S.R.
T.R.
1.0
0.8
0.6
0.4
0.2
0.0
2604 genes
Empty map
Iterative
SOM training
Final trained map
Transform to planar
surface for viewing
50
30
10
Gene A
Gene B
Gene C
Gene X
Gene Y
Gene Z

Fig. S12

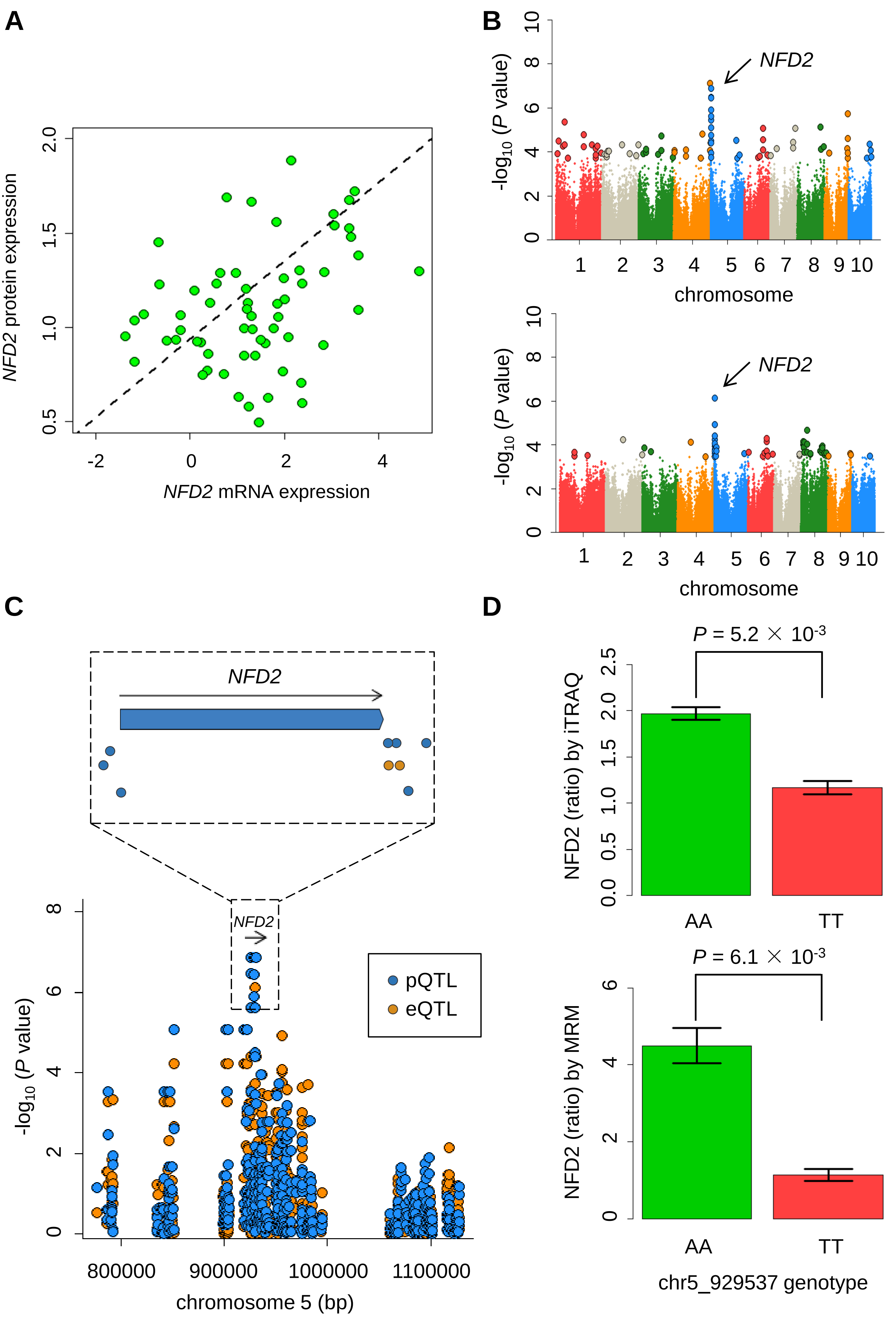

A
NFD2 protein expression
NFD2 mRNA expression
B
-log10 (P value)
NFD2
chromosome
C
NFD2
pQTL
eQTL
-log10 (P value)
chromosome 5 (bp)
D
P = 5.2 × 10-3
NFD2 (ratio) by iTRAQ
AA
TT
P = 6.1 × 10-3
NFD2 (ratio) by MRM
chr5_929537 genotype
